# The challenge of explicit learning in life skill education

**DOI:** 10.1038/s41539-025-00375-6

**Published:** 2025-12-01

**Authors:** Dominik M. Piehlmaier, Dee Warmath

**Affiliations:** 1 University of Sussex Business School, Brighton, UK; 2 Judge Business School, University of Cambridge, Cambridge, UK; 3 College of Family and Consumer Sciences, University of Georgia, Athens, GA USA

**Keywords:** Education, Information systems and information technology, Psychology, Psychology

## Abstract

Curriculum design in programs to build life skills often focuses on explicit learning methods that aim to increase declarative knowledge. However, this type of education has been shown to have minimal impact on behavior. We introduce a continuum of knowledge and argue that more flexible forms of knowledge are required to impact behavior, especially for novices. Using a randomized controlled trial conducted over several sessions, this study explores the ability of semi-flexible and flexible knowledge to promote optimal behaviors in the context of personal finances. We found that as knowledge became more flexible, desired changes in actual behavior became more likely. Our results provide evidence that life skills education programs, such as collegiate financial education, may be “barking up the wrong tree” with the focus on explicit learning. Expanding program design to incorporate a focus on flexible knowledge may improve the impact of such programs on desired behavior.

## Introduction

The efficacy of various learning strategies in higher education has received substantial attention in the literature^1–3^. However, comparability is hindered by the fact that performance is assessed differently depending on the subject at hand. For instance, while successful learning in applied fields, such as the life sciences, may be judged based on the ability to successfully execute certain tasks, more theoretical subjects, such as parts of the humanities, commonly assess learning based on objective knowledge (e.g., exams, quizzes, etc.)^3,4^. The spectrum of applied vs theoretical fields also includes subjects that are applied, but learning in higher education remains largely theoretical and, therefore, explicit. Take, for instance, the field of finance. While jobs in finance, such as product design, trading, or fund management, rely on the ability to assess situations and execute tasks, collegiate financial education is defined by the delivery of explicit knowledge^5^. Similarly, successful learning in a college-level finance course is commonly judged based on objective financial knowledge. This learning strategy is unproblematic as long as there is a causal link between increases in objective financial knowledge and optimal financial decision-making^6^.

Financial education has come under increased scrutiny since the Great Recession, with mixed findings^5,7,8^. In a recent study, Kaiser and colleagues^9^ provide evidence from a meta-analysis of 76 randomized controlled trials that tested various forms of financial education interventions. Their results suggest that financial education meaningfully improves financial literacy (usually tested using some form of financial knowledge quiz) and financial outcomes (saving, borrowing, insurance, budgeting, remittances) in the short-term; however, they found no reliable evidence of a lasting impact of such interventions. There has been a growing body of literature questioning whether financial education in its current form can achieve the desired outcome, namely a lasting improvement not only in financial literacy but also in the quality of financial decision-making^9–12^. For instance, Johan and colleagues^11^ report that a 14-week personal finance course did not improve financial attitudes or behavior among 512 Indonesian undergraduate students. Fernandes and colleagues^7^ did not find increases in positive financial behavior following financial education interventions. These results are in line with findings from Malaysia, India, and the US, among a range of other countries^10^.

A central issue may be that current collegiate financial education designs rely almost exclusively on building declarative domain-specific knowledge through explicit learning^13^. This form of knowledge can be considered factual knowledge, given the emphasis on what a person has stored in memory rather than their ability to apply it. Explicit learning is grounded in cognitive learning theory, which views learning as involving the organization, interpretation, and integration of information^14,15^. Bloom’s Taxonomy is one example of a cognitive approach to learning that lays out the categories of what students should learn^16^. Cognitive approaches to learning have been described as the “banking” approach to education in which the instructor deposits knowledge into the learner whose job is then to organize, interpret, and integrate that knowledge into their stores for future retrieval in a given situation^17^. In the case of financial education, such information includes financial concepts and calculations. For example, a financial education course might explain the principles or steps involved in budgeting and ask the students to complete an assignment in which they prepare a budget for a particular scenario. Cognitive or explicit learning lays a foundation of declarative knowledge, leaving it up to the individual to explore how to apply the knowledge beyond the instructor-designed and led examples covered during the learning process. While this approach to education has been shown to increase knowledge in the specific content taught^18^, it has not been found to produce improvements in the ability to reason an appropriate behavioral response in a given set of circumstances, especially for a novice^14^.

The status quo in collegiate financial education assumes that greater knowledge of the fundamentals of financial concepts and calculations or how financial markets work would lead to improvements in the capacity to make favorable financial decisions^8^. However, there is sufficient empirical evidence to challenge this assumption^10,11^. Take, for example, the integral financial concept of compound interest. Being able to calculate annuities will enable learners to answer questions regarding saving and borrowing. Yet being financially literate in this area does not directly translate into optimal borrowing or saving among consumers^8,10^. Actual financial decision-making involves an array of variables that consumers may find hard to align with necessarily simplistic present or future value calculations.

In learning theory, there is a recognition that learning is not the acquisition of declarative knowledge but the ability of the learner to adapt and apply that knowledge in a range of circumstances, some of which might not be envisioned by the instructor. Some suggest that this ability to apply learning tends to happen slowly over an individual’s life and experiences^19^. With experiential learning, there is recognition that “the purpose of education is to stimulate inquiry and skill in the process of knowledge getting, not to memorize a body of knowledge”^20^.

Experiential learning theory emphasizes the role of direct experience and reflection in the creation of knowledge and incorporates this exploration into the learning process itself, providing students with the freedom to take ownership of the knowledge gained and its application^20^. Here, the financial education course might ask students to develop a budget for themselves and reflect on their process after attempting to follow the budget over time. Research suggests that experiential learning methods tend to have a greater effect on behavior^21,22^. Yet, both cognitive and experiential learning methods tend to focus on declarative domain-specific knowledge (e.g., budgeting, saving).

Quite often, especially in financial education, declarative domain-specific knowledge tends to be factual or inflexible knowledge^23^. Factual knowledge involves memorizing and tends to deprioritize understanding of or ability to apply what has been learned^24^. In comparison, inflexible knowledge refers to basic or novice understanding of largely concrete, declarative concepts or information and the ability to execute such knowledge against a narrow set of domain-specific decisions or tasks^25^. For example, students who gain inflexible knowledge related to budgeting might be able to use their knowledge to make a budget in the way they were taught; however, they might struggle when circumstances require them to adjust the budget in ways that were not covered.

The problem with the emphasis on inflexible knowledge in collegiate personal finance courses is the applicability of the knowledge acquired^26^. Applicability issues arise from the fact that a three-month course cannot cover all (or even most) eventualities that a financial decision-maker will face, especially in a financial market that rapidly changes. Unlike other aspects of managerial education, foundational personal finance courses are not designed with experiential learning in mind^5^. Instead, they intend to introduce conceptual and methodological approaches in personal finance to increase financial literacy among learners. The acquired knowledge from such courses is partially inflexible because some real-world applications (i.e., financial decisions) are ill-structured, thus hindering the transferability of learned methods and concepts^27^.

It is possible, over considerable time and experience, for inflexible knowledge to become expertise through deliberate practice and reflection. This natural evolution involves mastery of domain-specific declarative knowledge, supported by the development of procedural and strategic knowledge. This expertise allows the decision-maker to adapt to unfamiliar circumstances within the original domain. For example, students of financial decision-making can gain expertise by applying the knowledge gained to ill-structured real-life financial decisions and learning from what happens next.

While experts are capable of applying their knowledge to a broad set of familiar and unfamiliar decisions, a defining feature of their application of knowledge tends to be a strategic approach to defining the problem or decision and constructing a response^28^. While this expertise can be developed through experience over time, our argument is that it can also be nurtured by what is taught. We ask the question: What if there was a way to encourage a more strategic approach to financial decision making without waiting for the estimated 10 years of deliberate practice to achieve expertise^29,30^? In the context of an individual’s financial life, 10 years can have a substantial impact on one’s financial well-being. Yet, there have been few attempts to explore innovative approaches to improve the impact of financial education at the collegiate level.

**Fig. 1:**
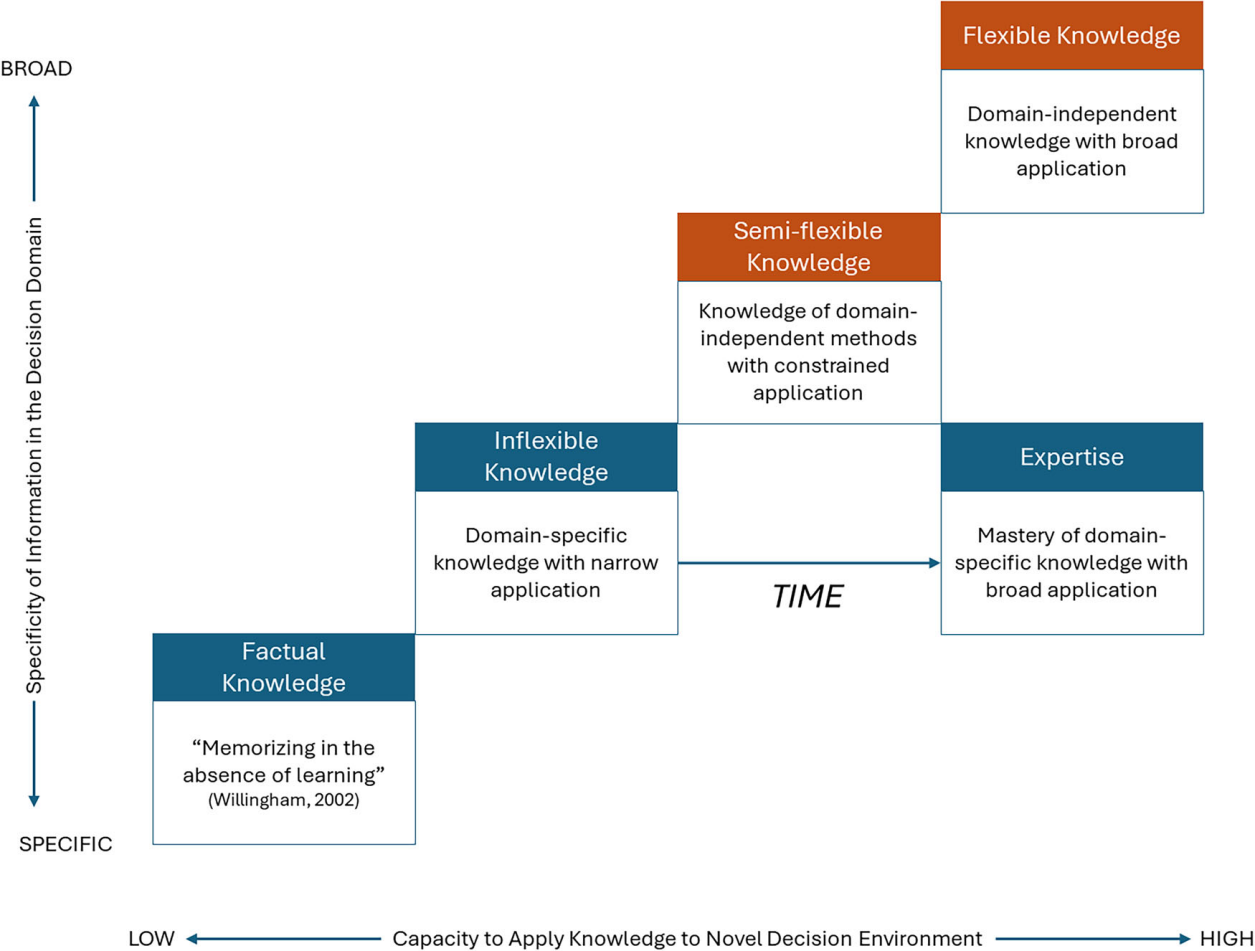
Continuum of knowledge. Note: The figure reflects the relationships between the types of knowledge included in the continuum. Blue boxes represent the current focus on financial education, and the orange boxes reflect the novel types of information being considered in this study.

In this paper, we introduce a continuum of knowledge incorporating two forms of domain-independent knowledge that might support better financial decision-making without waiting for financial expertise to manifest. One form of domain-independent knowledge is semi-flexible knowledge or knowledge of methods that transcend domains but are limited in the approach they offer. Statistical analysis methods are an example of semi-flexible knowledge. An individual might understand, for example, how to conduct a *t*-test or regression analysis across a variety of topics, but their application is narrow, given the assumptions and steps to apply the method. The other form of domain-independent knowledge is flexible knowledge that can be applied broadly across domains with few constraints. Strategic thinking (as a set of skills rather than theories^31^) is an example of flexible knowledge in that the insights and judgments learned can be applied broadly to familiar and unfamiliar problem solving and decision-making across life domains^28^. If we think of these forms of knowledge as a continuum, inflexible knowledge is the most basic and limited form, just one step ahead of factual knowledge. With factual knowledge, the individual struggles to apply what they know, even to decisions in the knowledge domain. Inflexible knowledge can be applied within the knowledge domain but struggles to transcend that domain. Semi-flexible knowledge allows for the knowledge to transcend a given domain, but limits the application to a specific method or approach. Flexible knowledge, which “can be accessed out of the context in which it was learned and applied in new contexts”^23^ is not limited to a given domain or method. Figure 1 depicts this continuum.

We use this continuum to argue that domain-independent forms of knowledge (i.e., strategic thinking and analytical methods) are required to achieve improvements in observable financial behavior for students enrolled in a collegiate financial education course. We focus on the latter due to its central importance to managerial education. Compared to previous studies that exclusively looked at traditional collegiate financial education with its transmission of inflexible knowledge^11^, the application of flexible and semi-flexible knowledge to financial education allows students to gain tacit or procedural knowledge that supports their ability to reason without necessarily increasing their declarative knowledge of the subject matter^14^. In this way, the focus on flexible knowledge mirrors elements of implicit learning related to the accumulation of procedural knowledge without the learner’s awareness of the subject matter or material at hand^32,33^. It is an integral part of business and managerial education, such as in strategy^31^ and leadership^34^ courses. Flexible knowledge provides learners with a tacit understanding or intuition of the right (or wrong) decision by informing the process or strategy of decision-making without relying on hierarchically structured knowledge^33^. We argue that this perspective leads to better behavioral choices (e.g., among saving, borrowing, and spending) in complex decision-making spaces, such as in finance^35^. In line with prior educational research, it is reasoned that “*the resulting knowledge is that of cues and conditions for choosing steps or the actions needed to solve a problem or to complete a task*”^18^.

Given the notable absence of flexible knowledge in collegiate financial education, there is a lack of empirical evidence to assess its impact on downstream financial behavior (e.g., an increase in saving rates). This study closes a gap in the literature by comparing the effectiveness of explicit collegiate financial education to strategic and analytical thinking education in producing positive financial behavior as measured by the selection of a market-optimal interest rate in comparison to immediate financial gratification (i.e., dominant intertemporal choice). We define dominant intertemporal choices as optimal financial decisions that optimize one’s financial outcome^36^. In the context of this study, these are choices that present students with the highest possible payment. The strategic thinking content seeks to enhance the individual’s ability to assess a situation, gain empathy for the needs present in the decision, and explore an alternative course of action through implicit knowledge gained throughout the course. Analytical thinking seeks to enhance the individual’s numeracy skills. This aspect addresses earlier reports that the inefficiency of financial education is caused by insufficient math skills^8^. Importantly, though, neither of the two courses in this study covers declarative knowledge in finance, only the traditional undergraduate-level personal finance course does. In other words, the former courses do not address financial decision-making or literacy in any shape or form.

Our objective is to determine whether there is causal evidence to suggest that collegiate financial education should consider flexible or semi-flexible knowledge in curricular design in order to address the issue of a missing link between collegiate financial education and positive financial behavior^11^. Our study also sheds light on the process that leads to higher financial literacy, improved financial behavior, or a combination of the two.

## Results

### Financial knowledge

**Fig. 2:**
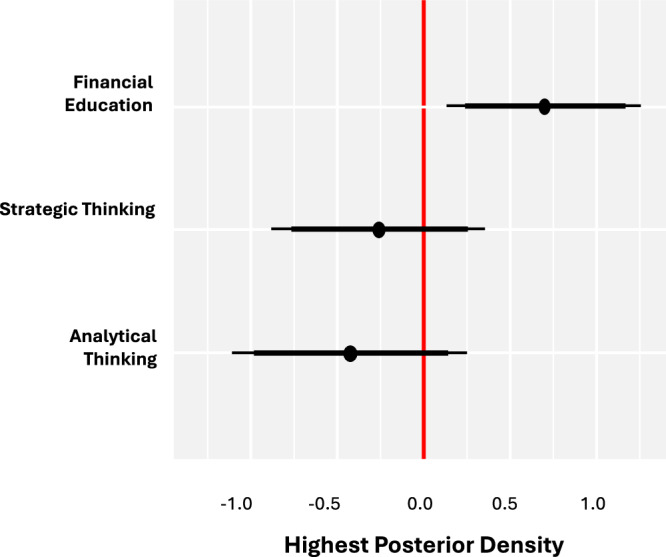
Financial knowledge treatment effects by group. Solid black dots indicate posterior means; bold black lines represent a 90% HPD; error bars indicate a 95% HPD.

**Fig. 3:**
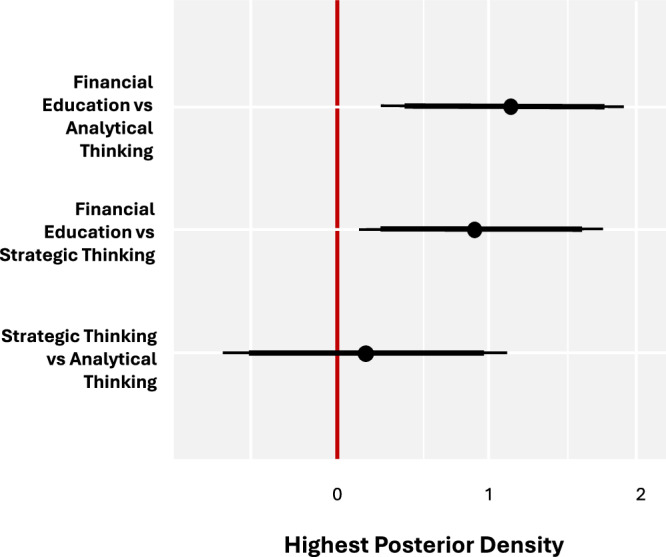
Comparison of group financial knowledge treatment effects. Solid black dots indicate posterior means; bold black lines represent a 90% HPD; error bars indicate a 95% HPD.

Using a randomized controlled design and Bayesian inference, we find that participants in the financial education arm were the only group to experience an increase in financial knowledge (posterior mean of initial vs subsequent percentage of correct answers to the financial knowledge quiz [pM]: 0.685, posterior standard deviation [pSD]: 0.302, 95% highest posterior density [HPD]: [0.09; 1.277]). The strategic thinking (pM: −0.249; pSD: 0.287) and analytical methods (pM: −0.436; pSD: 0.302) groups did not see any substantial impact on their financial knowledge from receiving the associated treatment. Figure 2 illustrates the treatment effects. The unsubstantial treatment effects for these two experimental arms were also reflected in the mean group differences (Fig. 3). Receiving five weeks of financial education improved financial knowledge compared to Strategic Thinking (ΔpM: 0.934; pSD: 0.418; 95% HPD: [0.114; 1.754]) and Analytical Methods (ΔpM: 1.121; pSD: 0.426; 95% HPD: [0.285; 1.962]). There was no detectable difference between the strategic thinking or analytical methods conditions (ΔpM: 0.188; pSD: 0.417). These findings are in line with prior research, and as expected, given that only the financial education group was taught explicit subject knowledge.

### Financial behavior in the form of intertemporal choice

Next, we examine the impact of the treatments on the intertemporal choice made. Table 1 contains the descriptive statistics showing the percent of participants selecting the immediate- and delayed-payment options. We used a set of Bayesian proportion tests for the evaluation of the results. The simple models applied Bååth’s^37^ objective priors and tested whether there were any detectable proportional differences between the treatment arms when it came to the selection of immediate vs delayed payments. We expected that strategic thinking and analytical methods would have a greater impact on financial behavior than education focused on explicit financial concepts. The convergence criteria of this simple model suggested that the following results can be deemed reliable. The Supplementary Information contains a graphical illustration of convergence.

**Table 1:** Percent of participants by payment timing

	Percent delayed ($54.00 in 5 days)	Percent immediate ($45.00 now)
Financial education	61.5	38.5
Strategic thinking	92.6	7.4
Analytical thinking	75.9	24.1

**Fig. 4:**
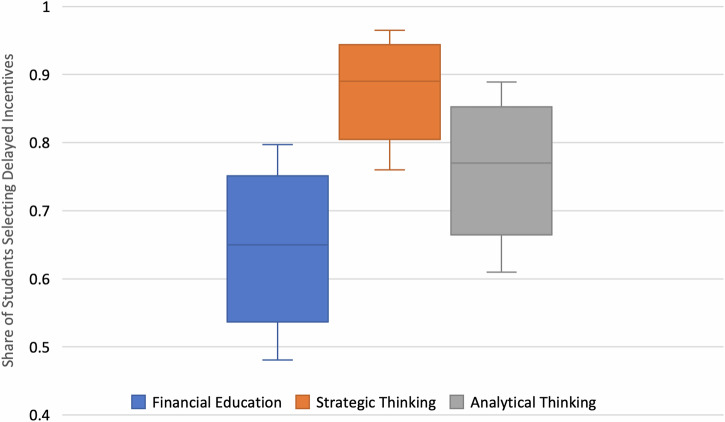
Boxplots for the posterior proportion of students selecting delayed incentives by group. Boxes range from the 25th to the 75th percentile with solid lines as medians and error bars as the 95% HPD.

**Fig. 5:**
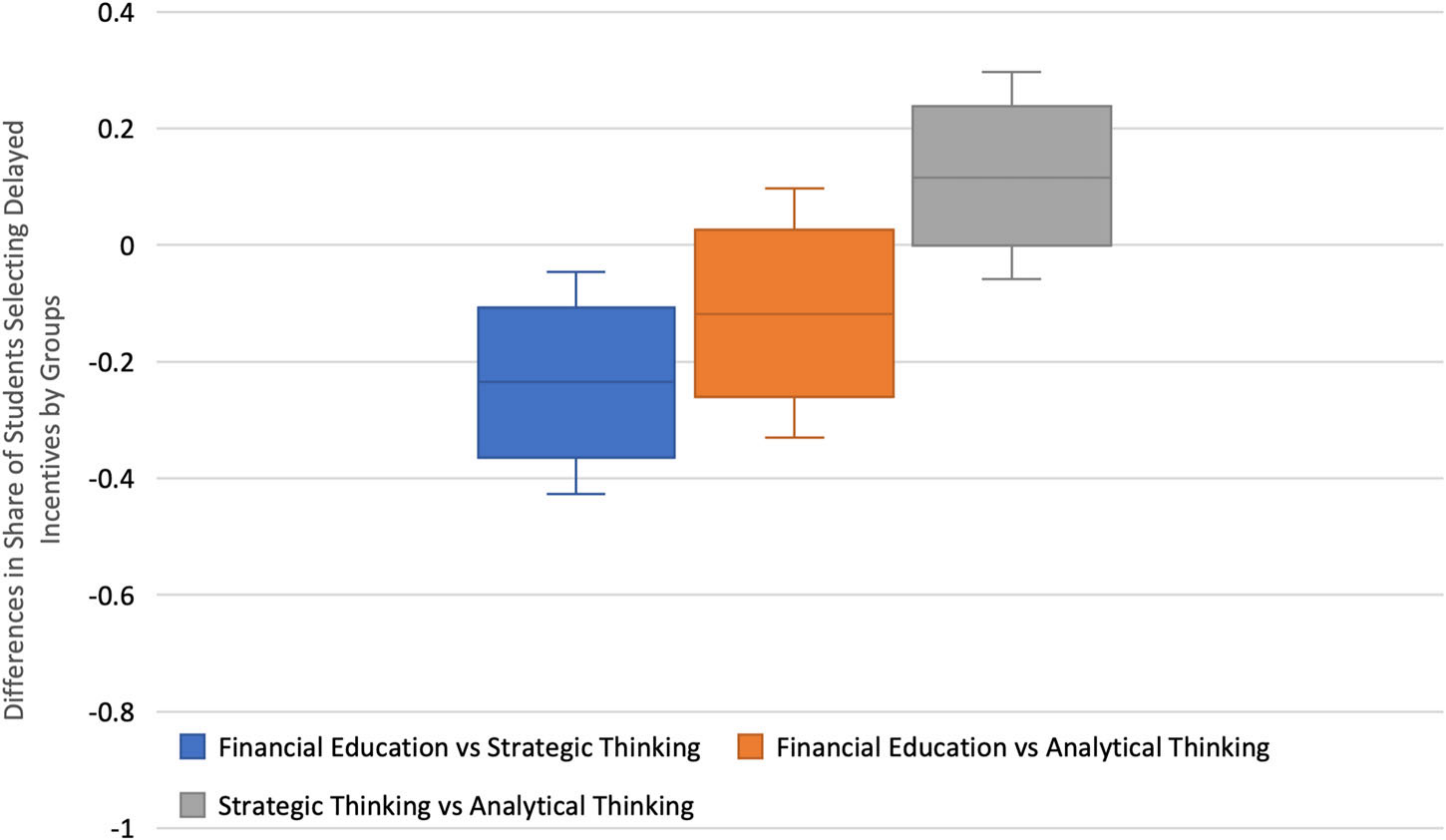
Boxplots for mean group comparison of the posterior proportion of students selecting delayed incentives. Note: Boxes range from the 25th to the 75th percentile with solid lines as medians and error bars as the 95% HPD.

Students in all three treatment conditions selected the delayed payment options more often than would be expected using a Laplace coinflip (i.e., better than chance; Fig. 4):

Financial education (pM: 0.647; pSD: 0.081), Strategic Thinking (pM: 0.883; pSD: 0.054), and analytical methods (pM: 0.765; pSD: 0.072). Comparing the three conditions in Fig. 5, we found that participants who were randomly allocated to the Strategic Thinking condition substantially outperformed their counterparts in the Financial Education arm (ΔpM: 23.6%; pSD: 9.8%; 95% HPD: [4.8%; 42.8%]; p(ΔpM > 0) > 99%). There was no statistically meaningful mean difference between explicit financial education and analytical methods conditions (ΔpM: −11.7%; pSD: 10.9%; 95% HPD: [−33.8%; 8.7%]; p(ΔpM < 0) < 86%) or between strategic thinking and analytical methods (ΔpM: 11.8%; pSD: 9%; 95% HPD: [−5.8%; 30.4%]; p(ΔpM >0) < 91%).

### Sensitivity analysis

Unlike a frequentist approach, our Bayesian estimations allow us to explicitly challenge the previously made assumption that none of the experimental treatments had any effect (i.e., the use of objective priors). A sensitivity analysis was conducted to test whether incorporating some statistical properties into our assumption would alter the reported findings. As mentioned, all presented results assume no prior information. In other words, null effects with large variance terms are assumed to reflect the high level of uncertainty. That said, one may argue that it is possible to restrict the range of feasible outcomes by considering the underlying scale. Specifically, some prior information could be incorporated into the models. More formally speaking, the applied objective priors essentially mimic a frequentist null hypothesis with means of zero and very low precision that require an infinitely large number of repetitions to stabilize the estimated mean effects. Yet it may be of interest to see the impact of a shift from objective to weakly informative priors that were readily available for the previously reported ANOVA approach. Given the underlying scales for financial knowledge, it was feasible to restrict the range of possible outcomes, which essentially limits the variability of the data to a sensible interval of observable outcomes. Therefore, the same unpooled Bayesian one-way ANOVA models were fitted with a truncated, instead of an unbounded, normal distribution. The limits for financial knowledge were set to range from −2 to 2.

This shift had virtually no impact on the reported outcomes for financial knowledge (explicit financial education pM: 0.686; pSD 0.3, Strategic Thinking pM: −0.251; pSD: 0.289, Probability pM: −0.44; pSD: 0.303). This indicates that, despite the relatively small in-group sample size, the illustrated results were robust against minor changes to the prior distribution. As a result, the main findings hold seemingly independent of whether objective or weakly informative priors are chosen*.* See Supplementary Information for an illustration of convergence.

## Discussion

As is true of many life skills education programs, collegiate financial education programs are currently designed to deliver declarative financial knowledge based on the belief that increased stores of such knowledge will lead to more favorable financial behavior and outcomes even among novices in a dynamic financial marketplace. The primary decision that designers of collegiate financial education in business schools believe they face is which financial concepts to include in the education. The knowledge imparted is factual (i.e., memorization without learning) or inflexible (i.e., domain-specific with narrow application), as would be expected in what is likely an early encounter with the financial content. There has been little work done to examine the role of other forms of knowledge or alternative educational approaches that are widely used elsewhere in higher education, including in other management and business courses^31,34^. Even with the financial concept knowledge approach, there has been little causal evidence for the effects of financial education. Using Bayesian estimation, we found that financial education in its current inflexible form produces declarative financial knowledge gains, although these gains may be short-lived^7^. However, compared to the domain-independent forms of semi-flexible and flexible knowledge (i.e., analytical method or strategic thinking), explicit learning of inflexible domain-specific knowledge has no traceable impact on the ability to make optimal intertemporal choices.

The experimental findings suggest that collegiate financial education is “barking up the wrong tree” in the sense that current designs of financial education produce improvements in financial knowledge that are not directly related to behavioral changes. In fact, the strategic-thinking content produced an intuition about financial decision-making that generated a substantially stronger improvement in recognizing optimal financial choice. Students receiving this flexible knowledge appeared better equipped to handle the procedural and strategic elements of financial decision-making than students who received education on financial concepts and calculations. Strategic or design thinking teaches students that the first step needs to be understanding the situation, identifying the needs or opportunities, and considering the connections between possible decisions or courses of action and desired outcomes.

The results have implications for the college student population and, more specifically, the development of targeted educational programs in business schools that can assist them with financial decision-making at this critical stage in their lives. One implication is the consideration of the objective of the education. Our findings are aligned with current literature suggesting that teaching students about topics such as debt, budgeting, investing, and savings produces improvements in tests of objective knowledge^38^. If building a store of domain-specific explicit knowledge is the objective of education, the current methods may be sufficient. However, if the objective of education is to support the individual in making better life decisions, our results suggest that the current methods are largely unable to deliver, at least in the near term^5,8,11^. Overall, these findings suggest that the design of life skills education programs (e.g., financial education) could be improved by teaching students how to frame the decision strategically. Our findings suggest that this might be accomplished by adding strategic thinking to the current explicit concept education (e.g., debt, budgeting, investing in the financial context). For example, financial education in business schools might incorporate lessons on conducting a situation analysis or the methods of design thinking (i.e., designing your financial life). There could be greater emphasis on what actions need to be taken or procedures need to be followed prior to making the financial decision or applying the financial concepts and calculations learned in the class. These tools are already part of most management-related disciplines and can be found in curricula in programs, such as project management and Master of Business Administration.

There are limitations to this study that should be considered. The experiment was conducted over a five-week period. Perhaps the effects of one or more treatment conditions on financial behavior or perceived financial decision-making ability take longer to manifest. A future study should consider additional follow-up periods to determine whether financial education delivered today influences financial behavior further down the road. Furthermore, this study contained one measure of financial behavior (i.e., the intertemporal choice) as an indicator of objective procedural knowledge. A future study could consider other measures. Lastly, the current study was conducted with 96 largely female, English-speaking, non-Hispanic White undergraduates from a single institution located in the American Midwest. Replications in other settings and with other demographics would provide valuable support or challenge the reported results.

From the perspective of this study, the real opportunity with collegiate life skills education is not which explicit concepts to teach. Instead, the debate should focus on the right balance between building stores of explicit financial concept knowledge and cultivating the ability to think strategically and holistically about consequential decisions. The current study provides a starting point for future research and discussion among scholars, educators, and policymakers.

## Methods

Using a randomized controlled trial conducted over several sessions at the University of Wisconsin-Madison, we tested two hypotheses. The first was whether collegiate financial education focused on explicit financial concepts produces a larger increase in declarative financial knowledge than strategic thinking or analytical methods. The second was whether instruction in strategic thinking or analytical methods produces more favorable financial behavior, measured as intertemporal choice, than education focused on explicit financial concepts.

### Participants

Ninety-six undergraduate students were recruited from the university’s paid subjects pool to provide feedback on recordings of actual lectures offered by the university in exchange for $45–$54 (see “Measures” section for details). Informed consent was obtained from all participants prior to the experiment. The study was performed in accordance with the Declaration of Helsinki and approved by the Education and Social/Behavioral Science Institutional Review Board of the University of Wisconsin-Madison (ID 20160263). Participants completing our study were between the ages of 19 and 25, with 77% being female (Table 2). As part of the screening criteria, they were undergraduate students who rated their own written and verbal English proficiency level as intermediate, advanced, or expert to ensure comprehension of educational material. We excluded students majoring in accounting or finance ex ante, as these students would have already received considerable education in financial concepts, which would have partially invalidated the financial education treatment.

**Table 2:** Characteristics of participants in the experiment

Characteristic	Percent of sample
Female	76.8
English as first language	75.6
White	65.9
Have a job	72.0
Sophomore standing	19.5
Junior standing	42.7
Senior standing	37.8

In total, 82 participants completed all requirements of the experiment. Thirteen dropped out of the study after the pre-survey and before the treatments began, and one dropped out after viewing the first set of lectures. The 14 incomplete cases were distributed across the treatment conditions (Table 3).

**Table 3:** Initial and completed participation by treatment

Treatment	Initial count	Completed count
Financial knowledge	32	26
Strategic thinking	32	27
Analytical thinking	32	29

Analyses of their pre-test responses were not different from the students who completed all requirements on financial knowledge and financial skill. Males were somewhat more likely to have dropped out than females (dropouts were 42.9 percent female; completes were 76.8 percent female). Consequently, there is evidence to suggest that missing at random is a reasonable assumption. Information on how missing data were handled can be found under “Analytical Approach”.

### Procedure

Three fictitious courses were created in the course management system of the university. Each course contained six prerecorded video lectures from existing undergraduate courses (Table 4). Explicit financial education lectures were taken from an existing course named Financial Capability and covered topics such as banking, investing, and budgeting. Strategic thinking lectures were taken from a design thinking/strategic planning course titled Consumer Strategy and Evaluation and covered topics such as strategic mission or objectives, design thinking, and reframing to define opportunities. The analytical methods lectures were taken from an applied statistics course titled advanced consumer analytics and included topics such as descriptive statistics, bivariate analysis, and regression. While the analytical methods course was not focused on the concept of numeracy per se, it did involve analytical thinking, working with numbers, and understanding probability from an accessible storytelling perspective.

**Table 4:** Topics covered by treatment and week

Treatment	Week	Topics
Financial education		
	Week 1	Planning your spending
		Banking
	Week 2	Savings
		Debt (Part 1)
	Week 3	Debt (Part 2)
		Retirement and investing
Strategic thinking		
	Week 1	The mission (intro)
		Design thinking
	Week 2	Gaining empathy and reframing
		Refining the idea
	Week 3	Telling your story
		Sharing your results
Analytical thinking		
	Week 1	Descriptive statistics
		The right tool
	Week 2	Crosstabs
		Correlations
	Week 3	*t*-tests
		Regression

Each fictitious course was constructed with only two assignments visible in each session. The functionality of the course management system allowed the turning on and off of access to each lecture series with the experimental schedule. This step prevented any participant from re-watching a previous lecture or skipping ahead to complete future lectures.

Students were recruited to the study ostensibly to provide feedback on the design of video lectures for university courses. Participants were randomly assigned to provide feedback on a given course (i.e., treatment condition) for the duration of the experiment. In addition, they were also randomly assigned to computers in the lab for each individual session. While different circumstances might lead us to view the lab as an artificial setting, these students spend a significant portion of their educational time viewing recorded lectures. Being asked to review content and provide feedback is part of their normal routine and encourages them to consider the content at a deeper level. This design also allowed us to avoid the issue of selection bias created by a comparison of students enrolled in an actual financial capability course to students who did not elect to enroll in such a course.

On each visit to the lab, a participant was handed a card with an alphanumeric code printed on it. This code was used as the participant’s identification number across the experimental sessions. Embedded in the code was an indicator of the treatment condition to which the participant was assigned. Random assignment was accomplished at the initial distribution of these cards by stacking them in repeating sequential order to achieve even, but random, assignment to treatment groups. Participants and study administrators were unaware of the course assignment. Randomized seat assignment for each visit was accomplished by having participants draw a number from a shuffled deck to indicate which computer terminal to use.

During the first visit, all participants completed the same pre-survey containing measures of financial knowledge. In weeks 2 through 4, participants entered the lab, obtained their identification card, found the computer to which they were assigned, logged into the course management system, put on their headphones, and listened to their assigned lectures. Two activities encouraged participant engagement with the lecture material. First, participants were instructed to take notes, as well as write a summary of what they had learned and include any questions they had from viewing the lecture. After each lecture, participants completed a brief, self-paced online survey to collect feedback on the material presented in order to ensure engagement. In week 5, participants returned to complete the post-survey that was identical to the survey they completed in week 1. The study concluded with a debrief.

### Measures

The outcome measures for this study reflect the proximate and ultimate intended outcomes of financial education—financial knowledge and the ability to engage in optimal financial behaviors. We measured financial knowledge using the 13-item scale developed by Fernandes, Lynch, and Netemeyer^7^. This scale includes objective knowledge questions, such as “*Do you think that the following statement is true or false? After age 70 1/2, you have to withdraw at least some money from your 401(k) plan or IRA.”*—or—*“When an investor spreads his money among different assets, does the risk of losing a lot of money increase, decrease, or stay the same?”* We used the percentage of the 13 items answered correctly by the individual as our measure of financial knowledge.

The individual’s financial behavior was measured by their selection of immediate vs delayed incentive payments. Participants were offered $54 (20% more) if they were willing to wait five days for payment. If they refused the option, they were paid $45 for completing the five 1-h sessions. The $9 increase is equivalent to a 1460% annual interest rate, which was the dominant intertemporal choice given the fact that it vastly outperforms any sufficiently risk-free market option at the time of the data collection. We used a binary variable coded as one if they decided to wait for the $54 (i.e., selecting the dominant intertemporal choice) and zero if they decided to take the $45 immediately.

### Analytical approach

Predictive mean matching (PMM), a semiparametric multiple imputation chain equation (MICE) method (seed: 1234), using the fifth nearest neighbor, was applied to impute 10 datasets in order to account for the 14 dropouts from pre- to post-test condition. The procedure was executed in the R package MICE^39^ using a complete set of auxiliary variables in order to guarantee reliable results. A randomly drawn imputed dataset (#1) was used for the subsequent Bayesian estimations that are described below^40^. The random draw bypasses the issue around pooling posterior distributions post estimation that is commonly resolved by augmenting data, a slow and statistically inefficient procedure^41^.

The aim of our analyses was to derive fully transparent and replicable results while avoiding some of the more common obstacles of experimental research, e.g., small sample sizes adjustments, restrictive normality and independence assumptions, etc.^42^ Bayesian inference was chosen due to its ability to account for the inherent uncertainty that is connected with the novel experimental setting. In addition, the approach is able to handle the relatively small in-group sample size. It can also utilize the availability of sensible weakly informative priors due to the underlying standardized scales, which offered clearly defined cutoff points that could be used to construct a truncated normal distribution to challenge the applied objective priors. This approach allowed us to forego any frequentist small sample size adjustments while obtaining robust estimates that were sufficiently independent of prior assumptions. In essence, our Bayesian approach retains the interpretability while avoiding some of the aforementioned limitations of commonly used frequentist inference.

All models were implemented in R using Gibbs sampling within the rjags package^43^. Our expectations were tested with an unpooled Bayesian one-way analysis of variance (ANOVA) that allows for intergroup heteroskedasticity. The models utilized three Markov chain Monte Carlo (MCMC) algorithms with 1000 iterations each for adaptation and burn-in, 100,000 iterations to monitor, a thinning interval of 10, and a random seed of 1234. Convergence is discussed prior to presenting the results, and the impact of the applied objective priors is challenged in a subsequent sensitivity analysis. The intertemporal question was assessed using a set of Bayesian proportion tests with three MCMC chains, 1000 adaptive iterations, no thinning, and 5000 iterations to monitor, using Bååth’s broad priors^37^.

To assess the ability of explicit financial concept education, strategic thinking, and analytical thinking to produce improvements in financial knowledge, we fitted a set of Bayesian one-way ANOVA models that were robust against heteroskedasticity. Equality of variance is an overly restrictive assumption that is not supported by the underlying data; therefore, our model is more appropriate to examine the three experimental arms with heteroskedastic error terms. Normally distributed objective priors with mean zero and a precision (i.e., inverse variance) of 0.0001 were applied. This low precision accurately reflects the high level of uncertainty that is connected with the novel experimental setting. The residual precision, τ, was modeled to follow a gamma distribution with uniformly distributed hyperparameters for rate and scale ranging from 0 to 1. The initial Bayesian estimation aimed to test for potential group differences and treatment effects between participants who were previously exposed to explicit financial education, strategic thinking, and analytical methods lectures. The model exhibited no noticeable autocorrelation after the first few iterations, a desirable level of state changes as the chains walked, sufficiently smooth density plots, and good overall mixing. This strongly indicates that the algorithm converged to its stationary distribution. The Supplementary Information contains further details. Consequently, the presented results were deemed reliable.

## Supplementary information

Supplementary information

## Data Availability

Data are available from the authors upon reasonable request.

## Code availability

The code to derive the reported results was written in R Studio and is available from the authors upon reasonable request.
