# Supplementary material for: The challenge of explicit learning in life skill education
Source: NPJ Sci Learn. 2025 Dec 1;10:87. doi: 10.1038/s41539-025-00375-6 (PMC12669786; doi:10.1038/s41539-025-00375-6)
Supplement: Supplementary file 1 — Supplementary information [file 41539_2025_375_MOESM1_ESM.pdf]

SUPPLEMENTARY INFORMATION

THE CHALLENGE OF EXPLICIT LEARNING IN LIFE SKILL EDUCATION

Table of Contents

Financial Knowledge Objective Priors 1
Financial Knowledge Sensitivity Analysis 4
Perceived Financial Self-Efficacy Objective Priors 9
Perceived Financial Self-Efficacy Sensitivity Analysis 13
Bayesian Proportion Tests for Choice of Immediate vs Delayed Payment 17

Financial Knowledge Objective Priors

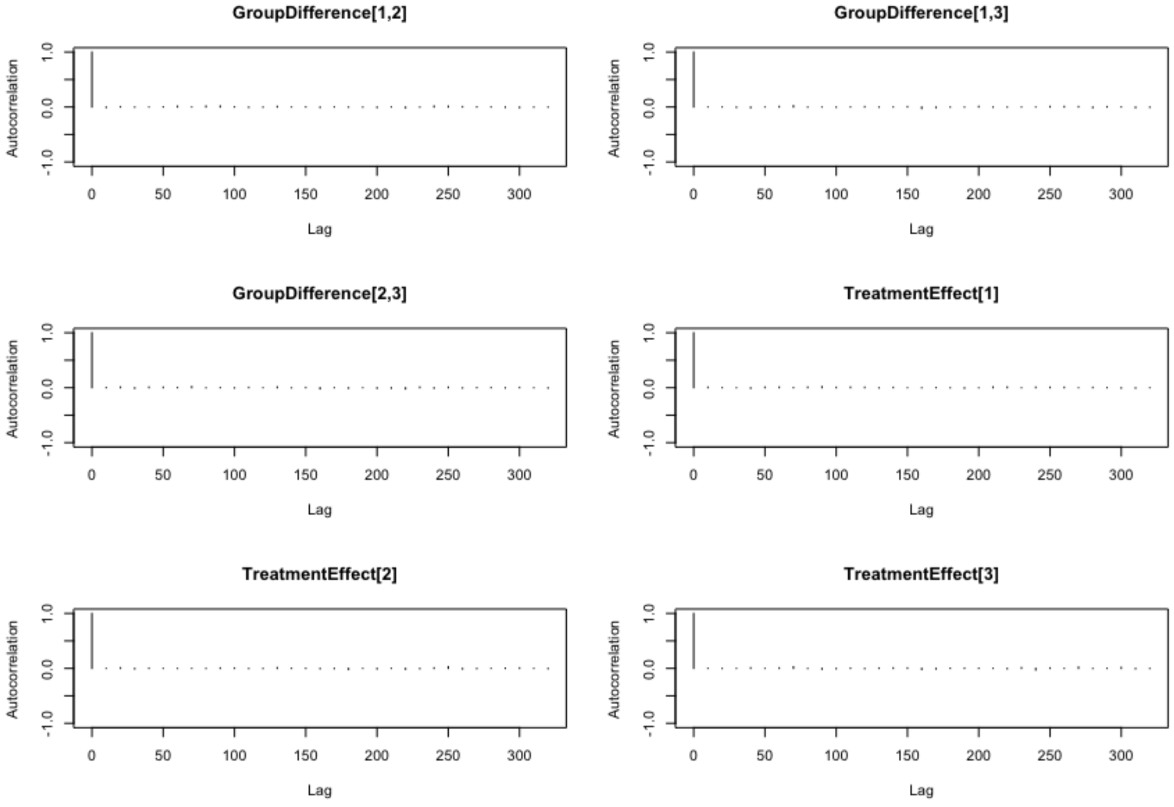

Supplementary Figure 1: Financial Knowledge Objective Priors Autocorrelation Plots

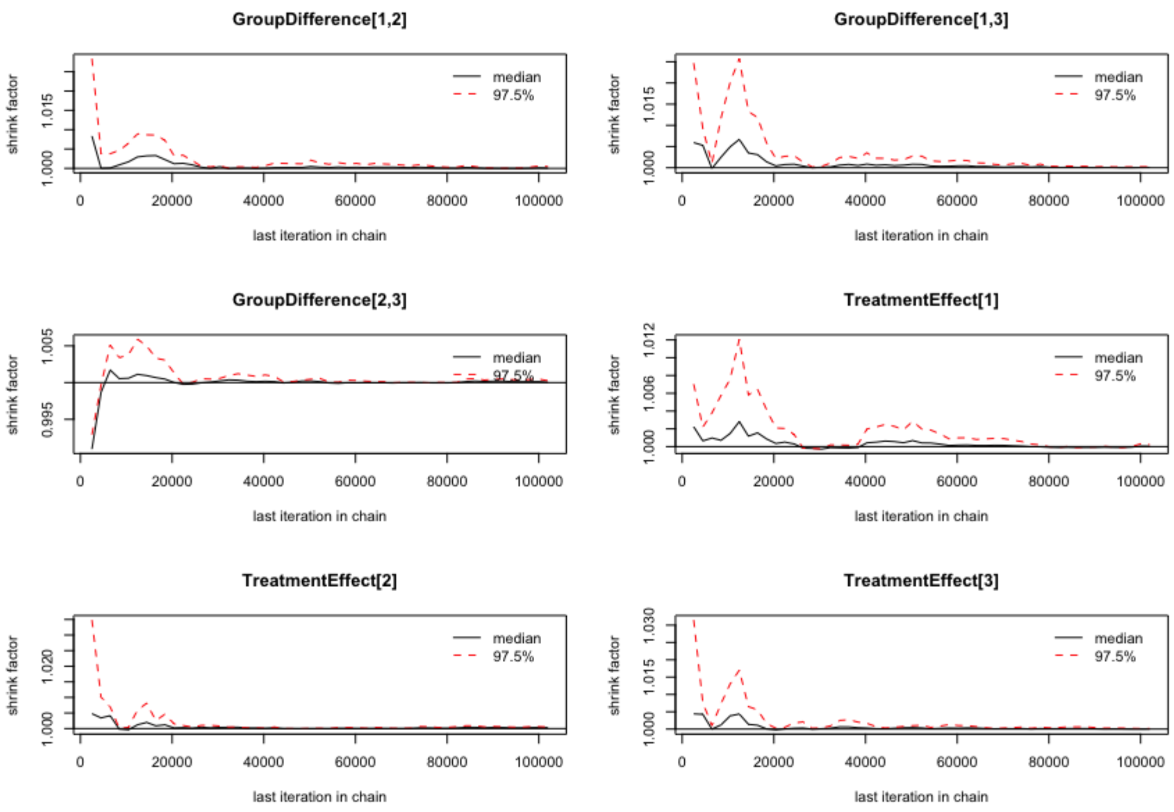

Supplementary Figure 2: Financial Knowledge Objective Priors Gelman Plots

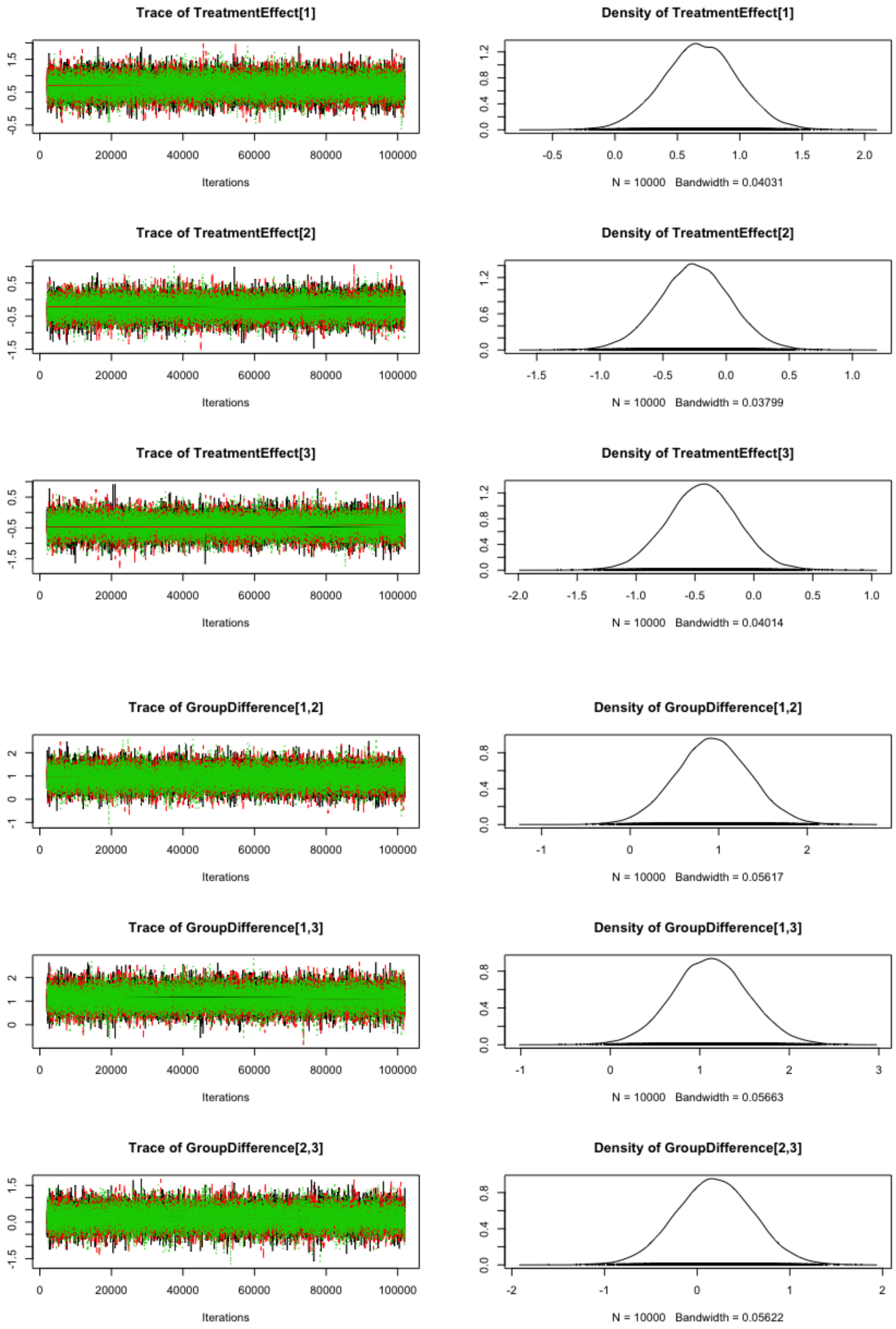

Supplementary Figure 3: Financial Knowledge Objective Priors Trace and Density Plots

Financial Knowledge Sensitivity Analysis

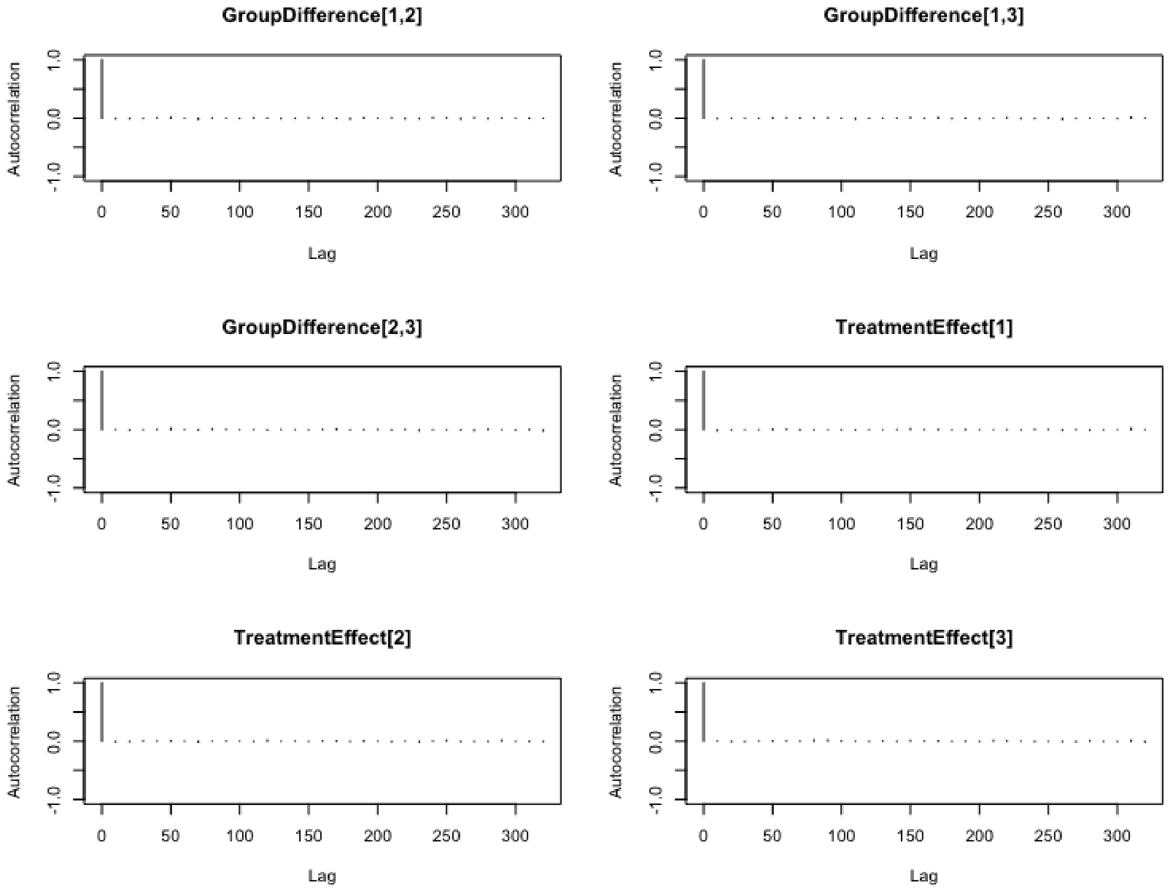

Supplementary Figure 4: Financial Knowledge Sensitivity Analysis Autocorrelation Plots

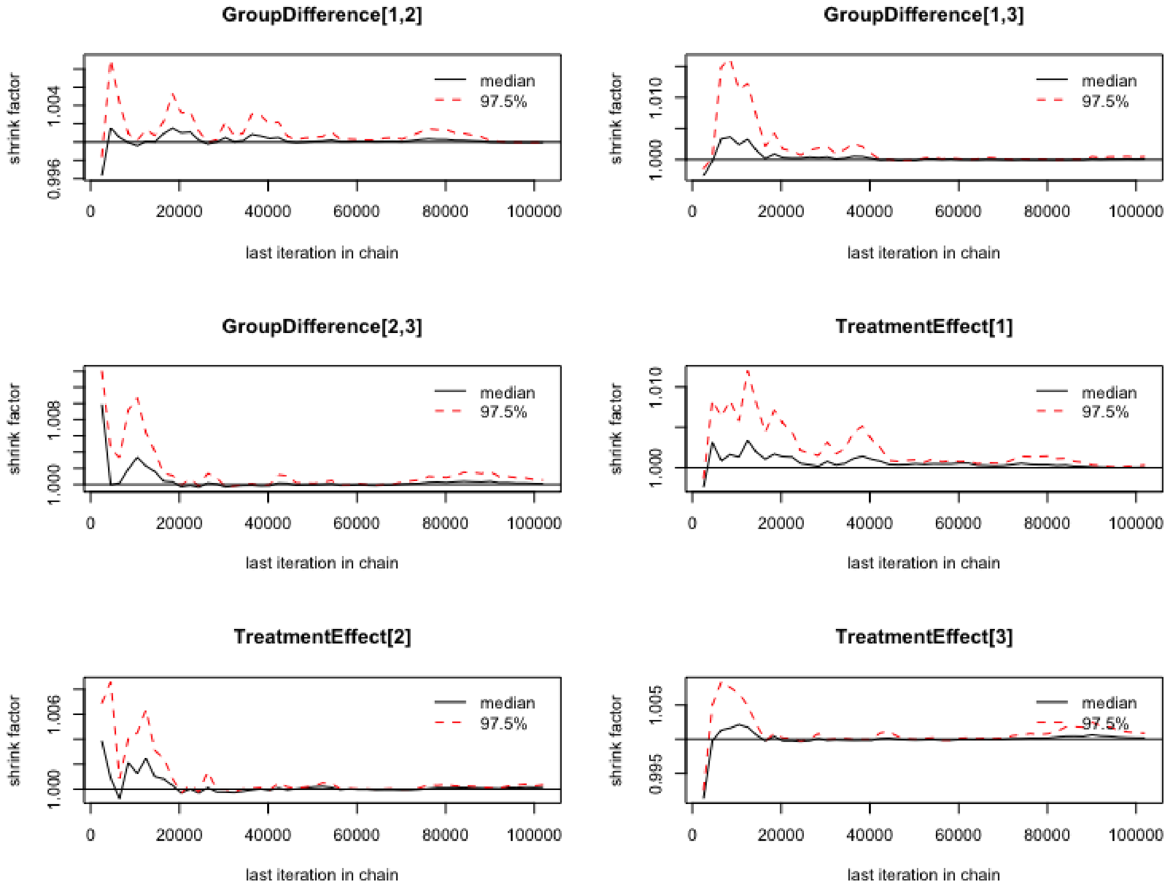

Supplementary Figure 5: Financial Knowledge Sensitivity Analysis Gelman Plots

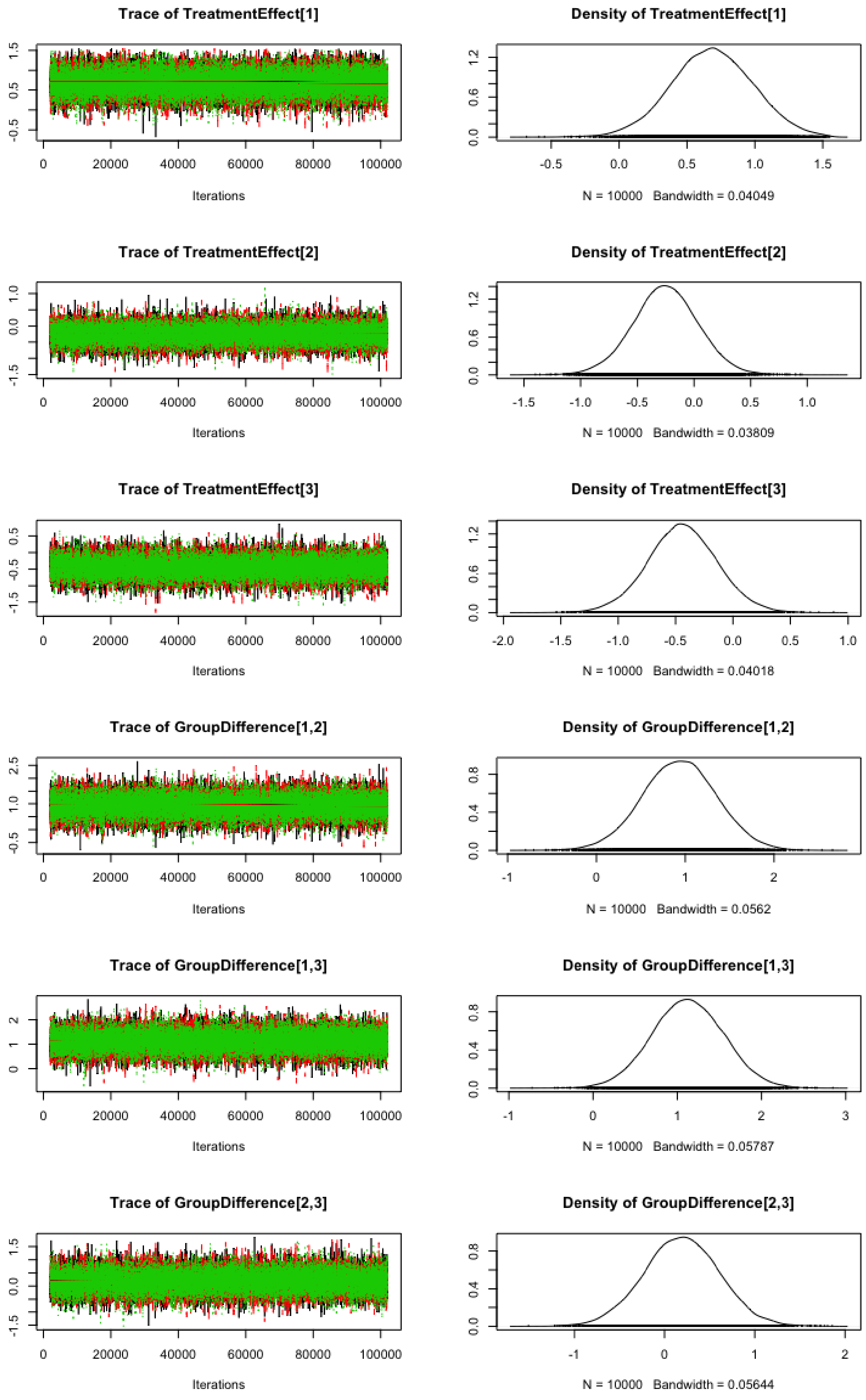

Supplementary Figure 6: Financial Knowledge Sensitivity Analysis Trace and Density Plots

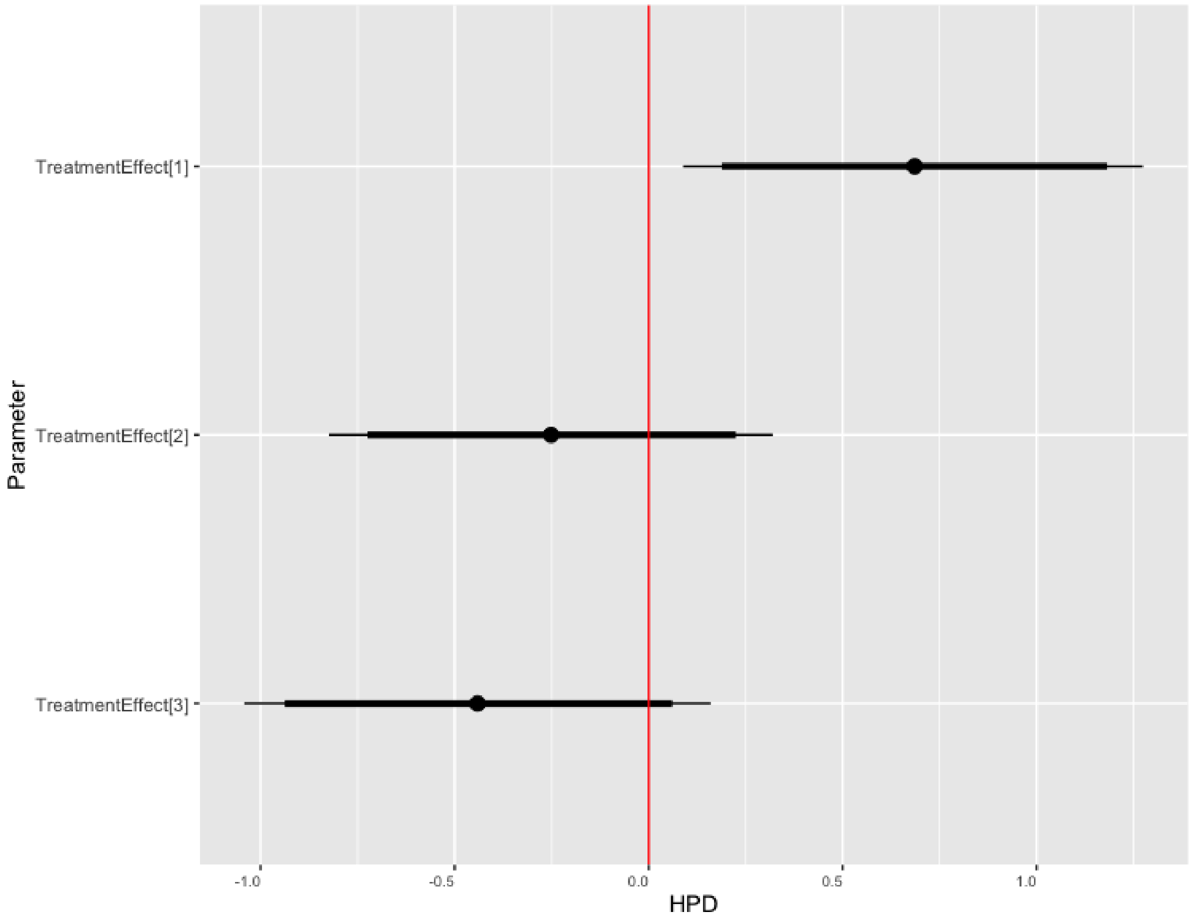

Supplementary Figure 7: Financial Knowledge Sensitivity Analysis Treatment Effects by Group
Note: Group [1]: Financial Education; [2]: Design Thinking; [3]: Numeracy
HPD: Highest Posterior Density; solid black dots indicate posterior means; bold black lines represent a 90% HPD; error bars indicate a 95% HPD

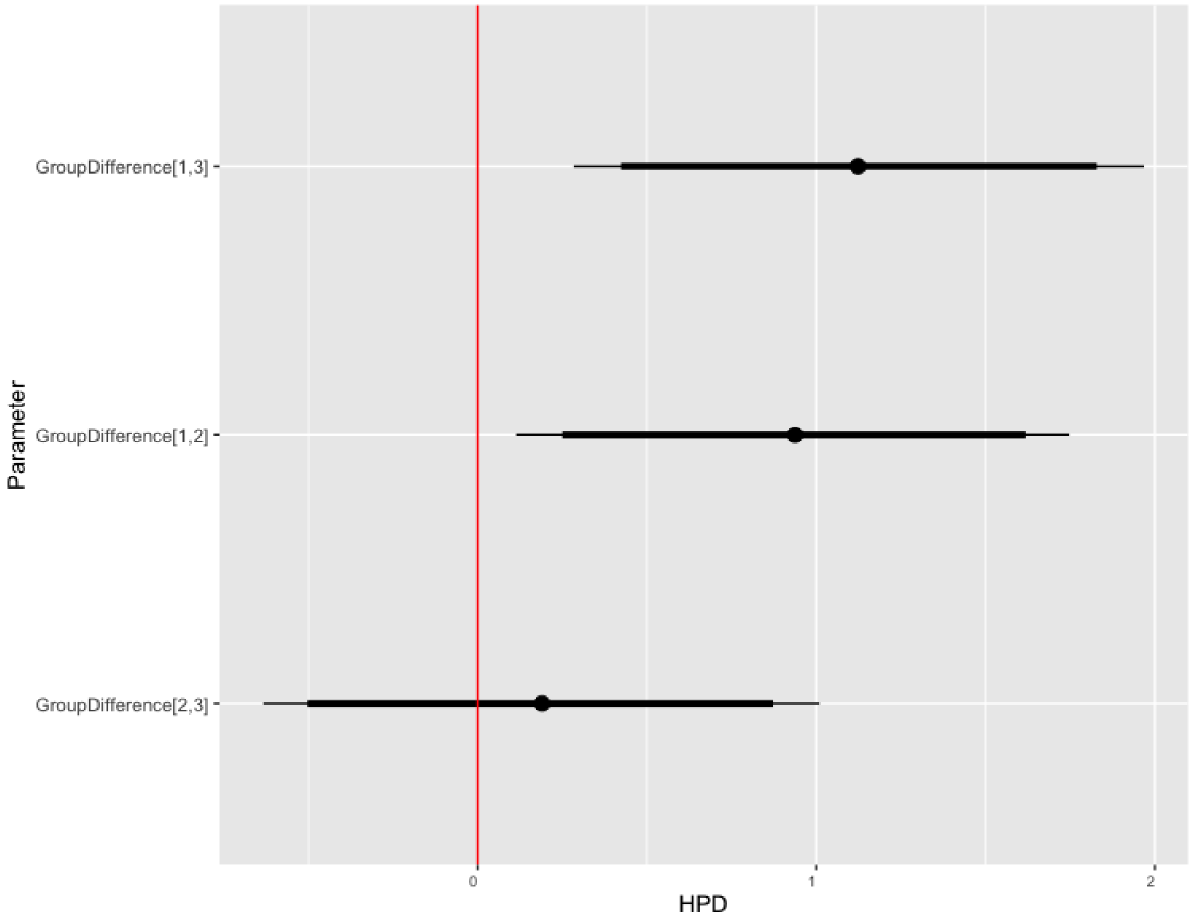

Supplementary Figure 8: Financial Knowledge Sensitivity Analysis Group Differences
Note: Group [1]: Financial Education; [2]: Design Thinking; [3]: Numeracy
HPD: Highest Posterior Density; solid black dots indicate posterior means; bold black lines represent a 90% HPD; error bars indicate a 95% HPD

Perceived Financial Self-Efficacy Objective Priors

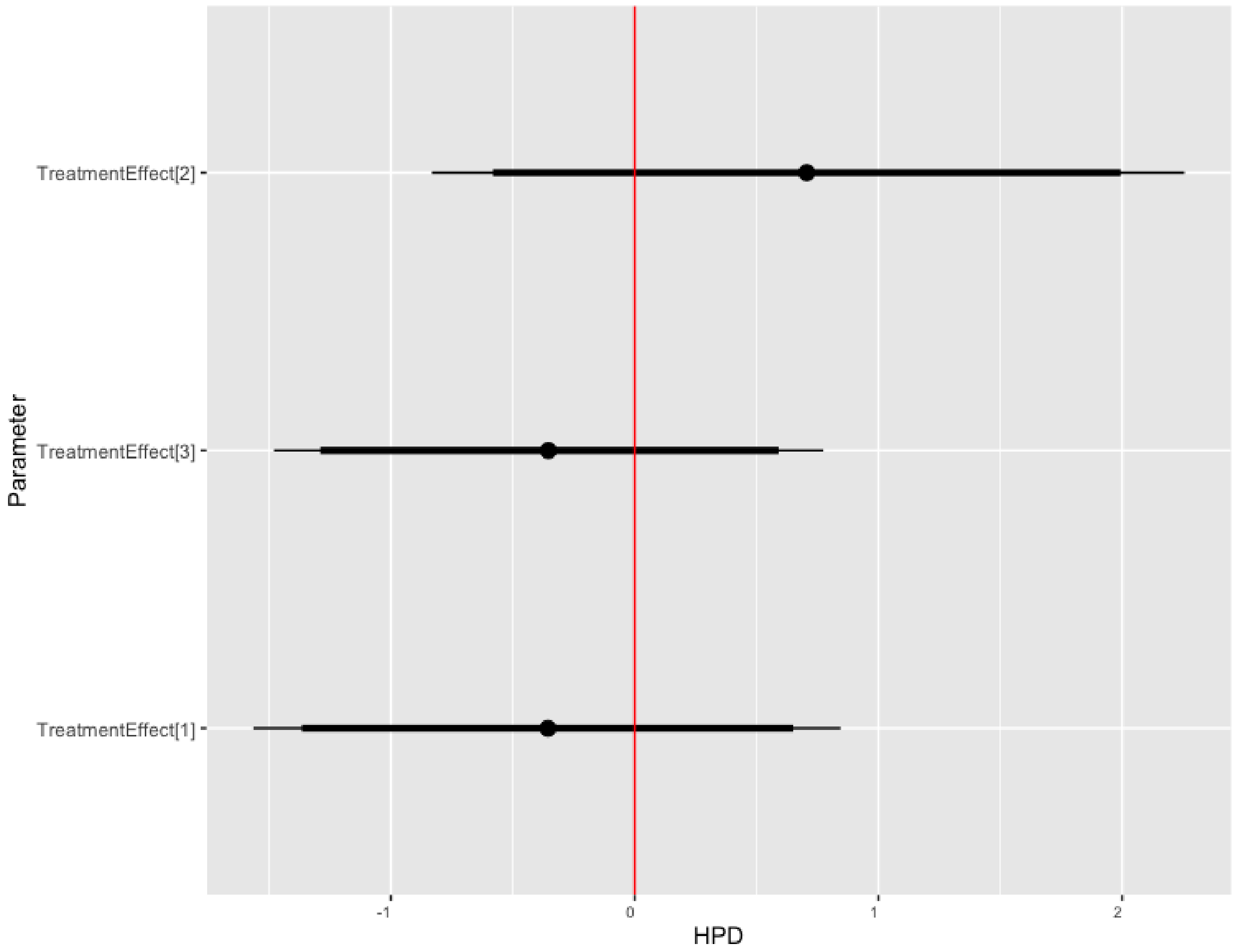

Supplementary Figure 9: Perceived Financial Self-Efficacy Treatment Effects by Group
Note: Group [1]: Financial Education; [2]: Design Thinking; [3]: Numeracy
HPD: Highest Posterior Density; solid black dots indicate posterior means; bold black lines represent a 90% HPD; error bars indicate a 95% HPD

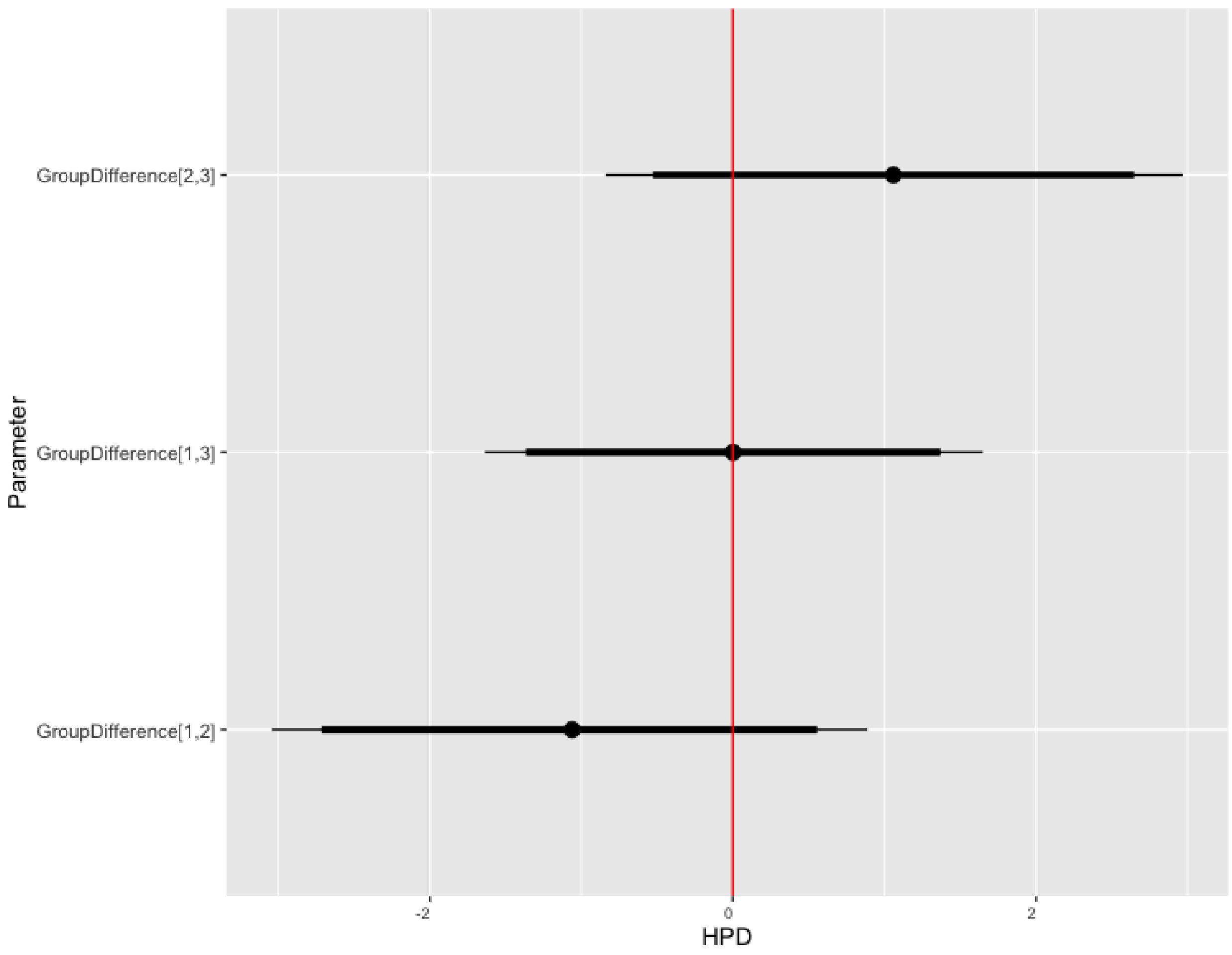

Supplementary Figure 10: Perceived Financial Self-Efficacy Group Differences
Note: Group [1]: Financial Education; [2]: Design Thinking; [3]: Numeracy
HPD: Highest Posterior Density; solid black dots indicate posterior means; bold black lines represent a 90% HPD; error bars indicate a 95% HPD

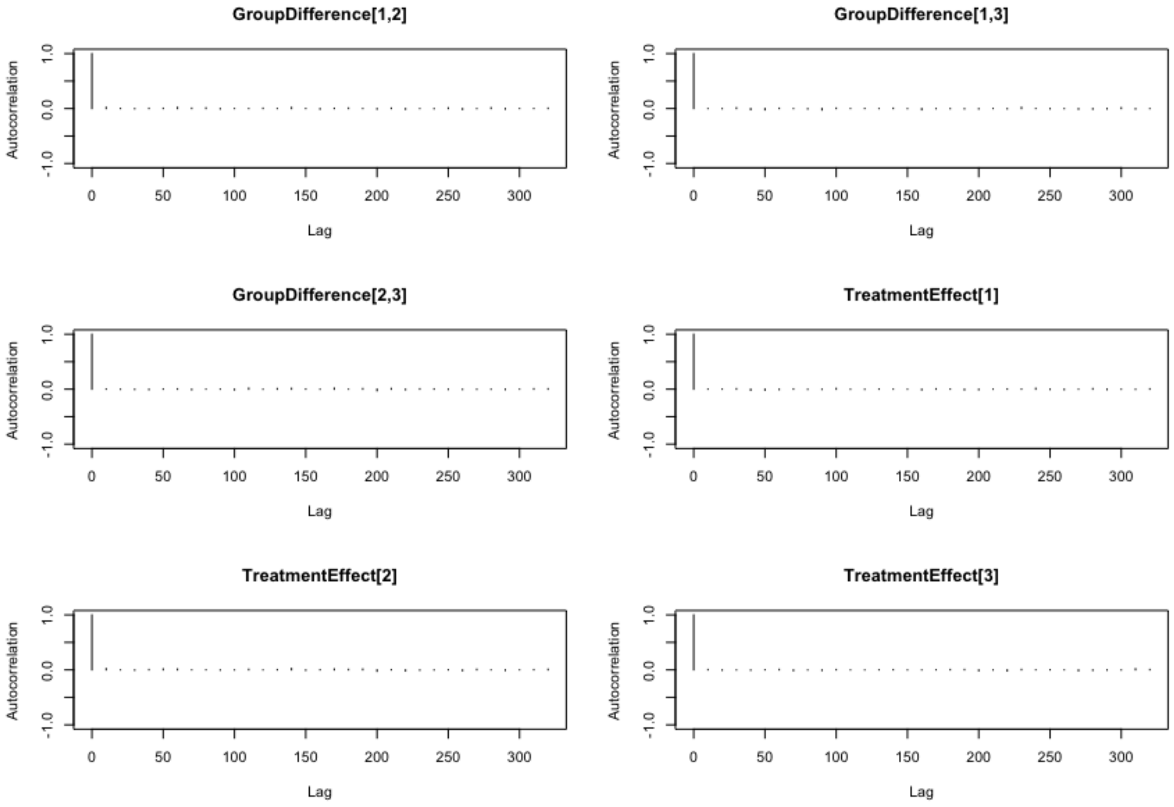

Supplementary Figure 11: Perceived Financial Self-Efficacy Objective Priors Autocorrelation Plots

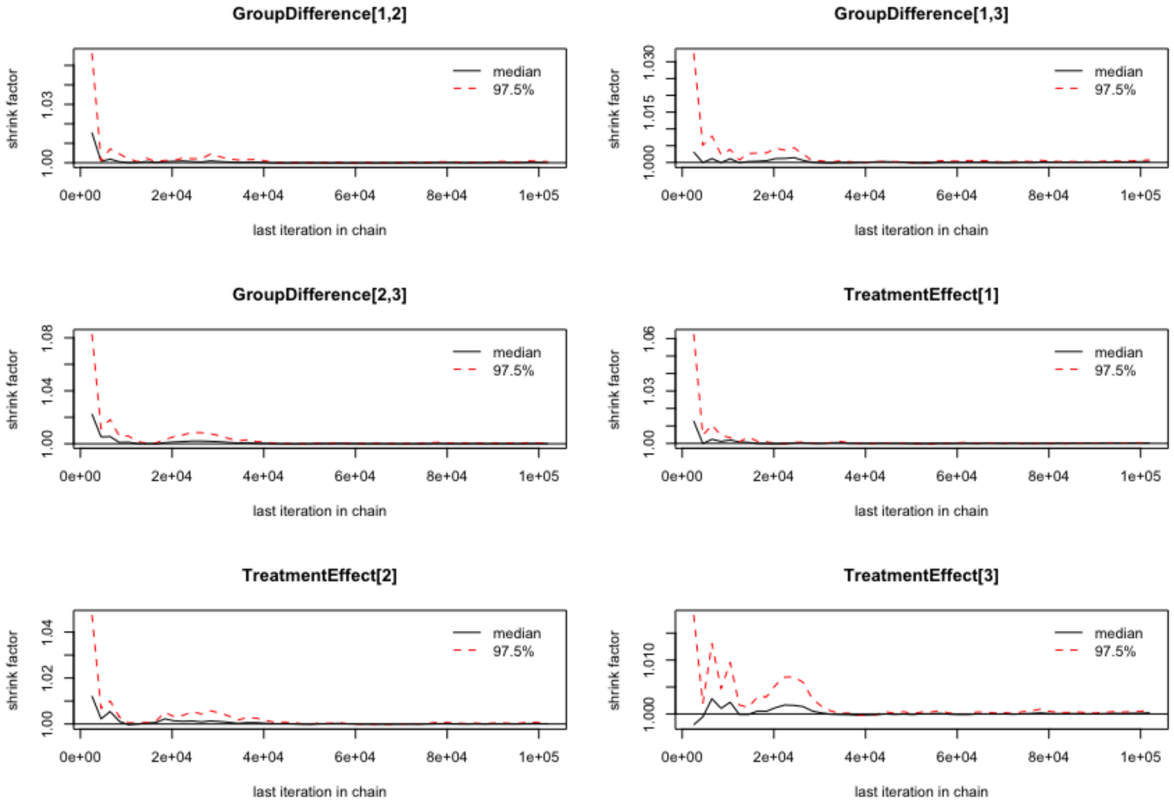

Supplementary Figure 12: Perceived Financial Self-Efficacy Objective Priors Gelman Plots

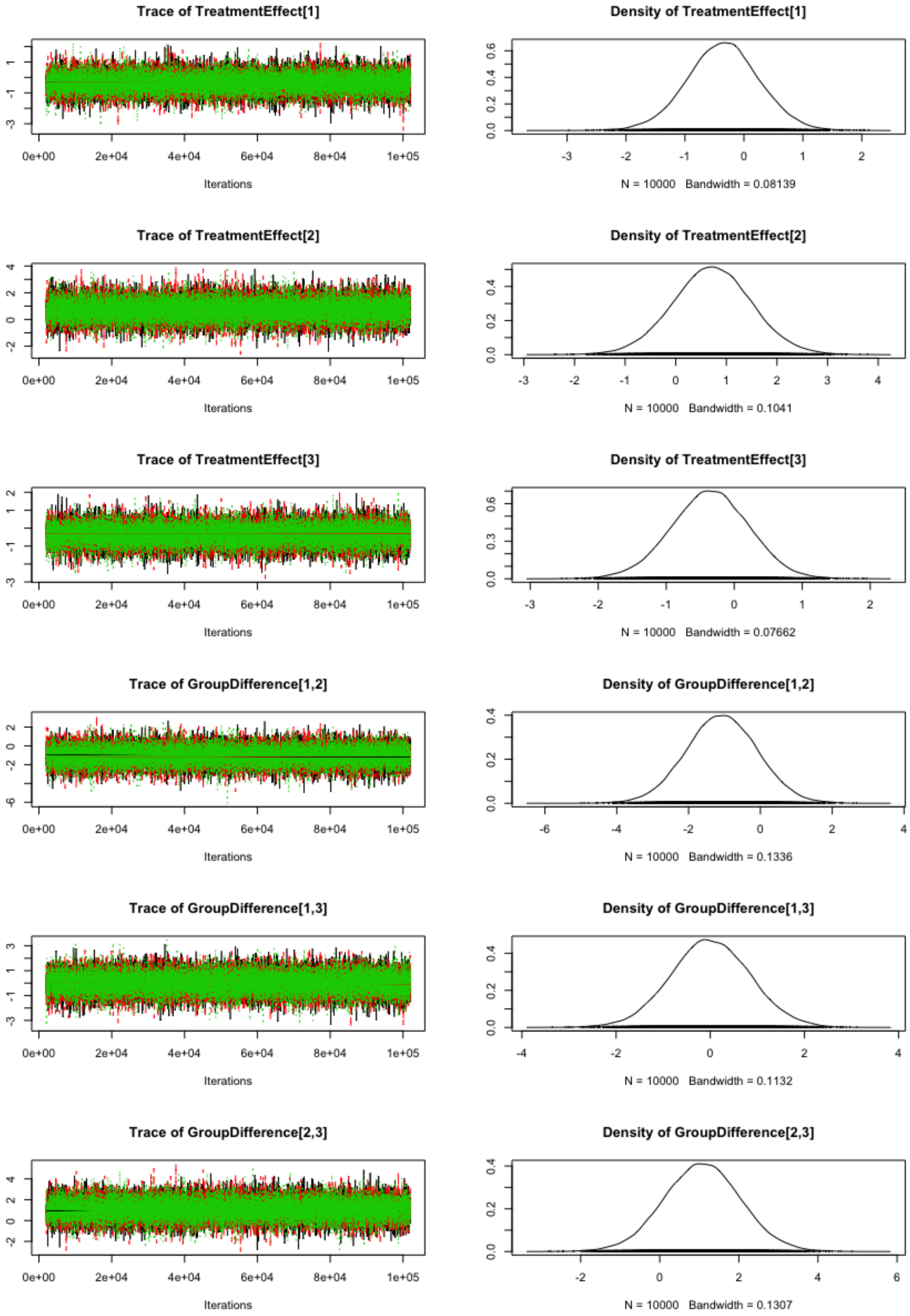

Supplementary Figure 13: Perceived Financial Self-Efficacy Objective Priors Trace and Density Plots

Perceived Financial Self-Efficacy Sensitivity Analysis

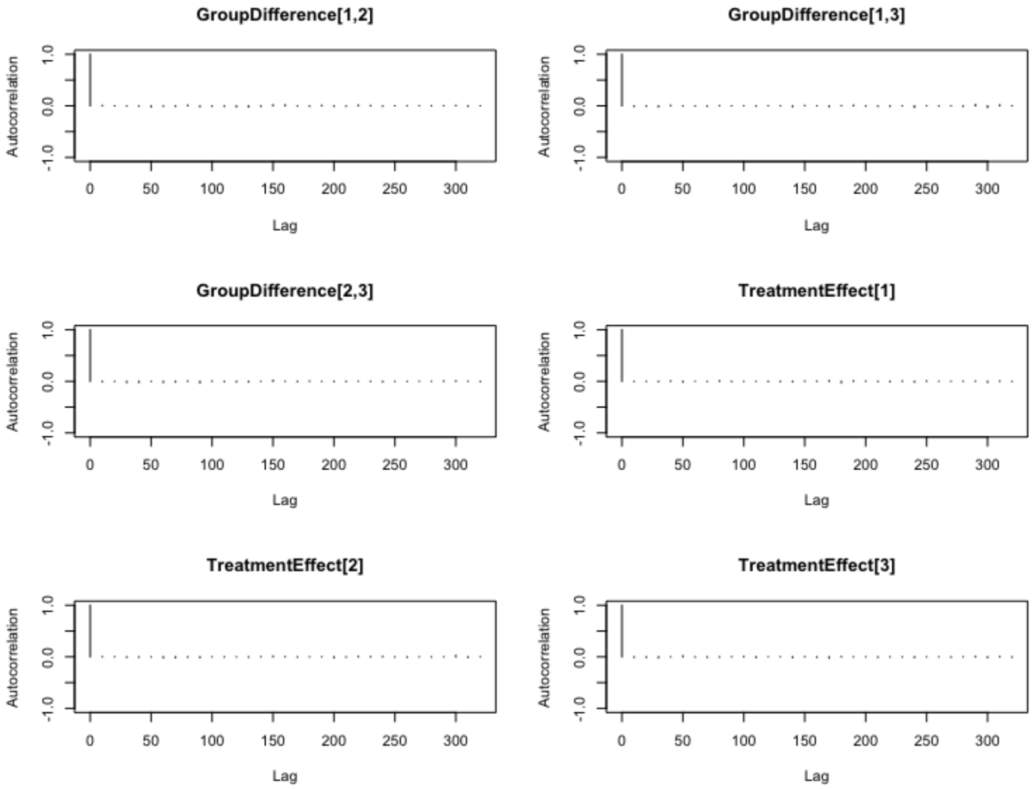

Supplementary Figure 14: Perceived Financial Self-Efficacy Sensitivity Analysis Autocorrelation Plots

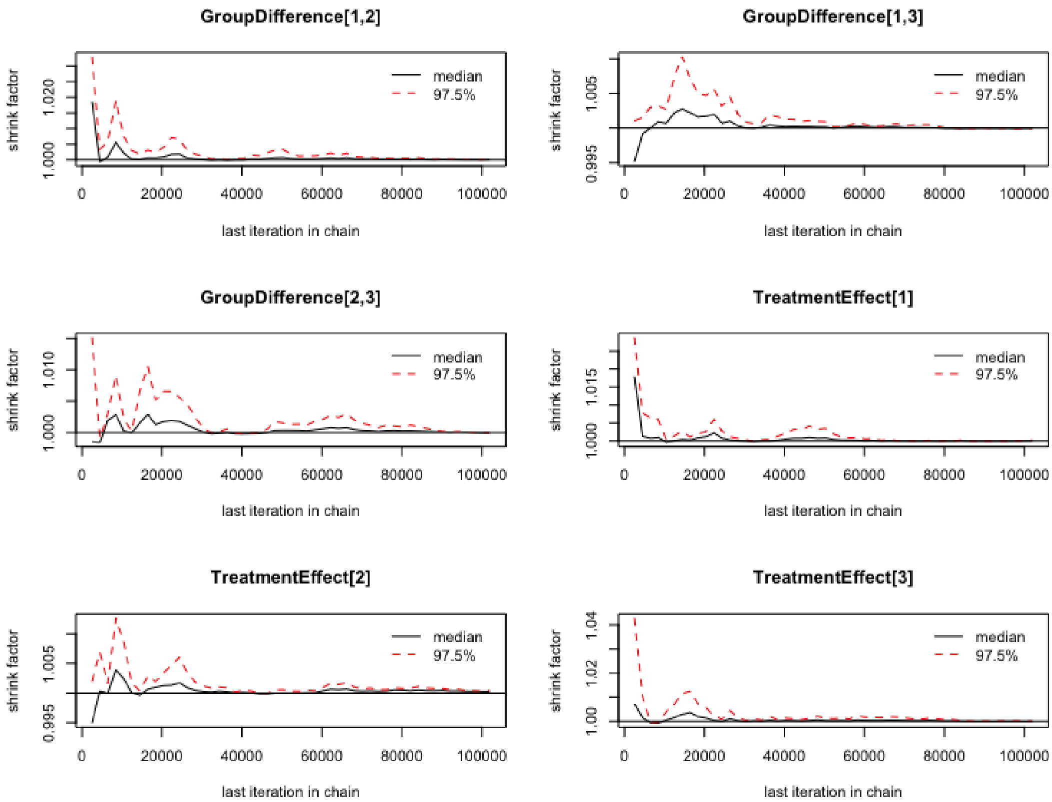

Supplementary Figure 15: Perceived Financial Self-Efficacy Sensitivity Analysis Gelman Plots

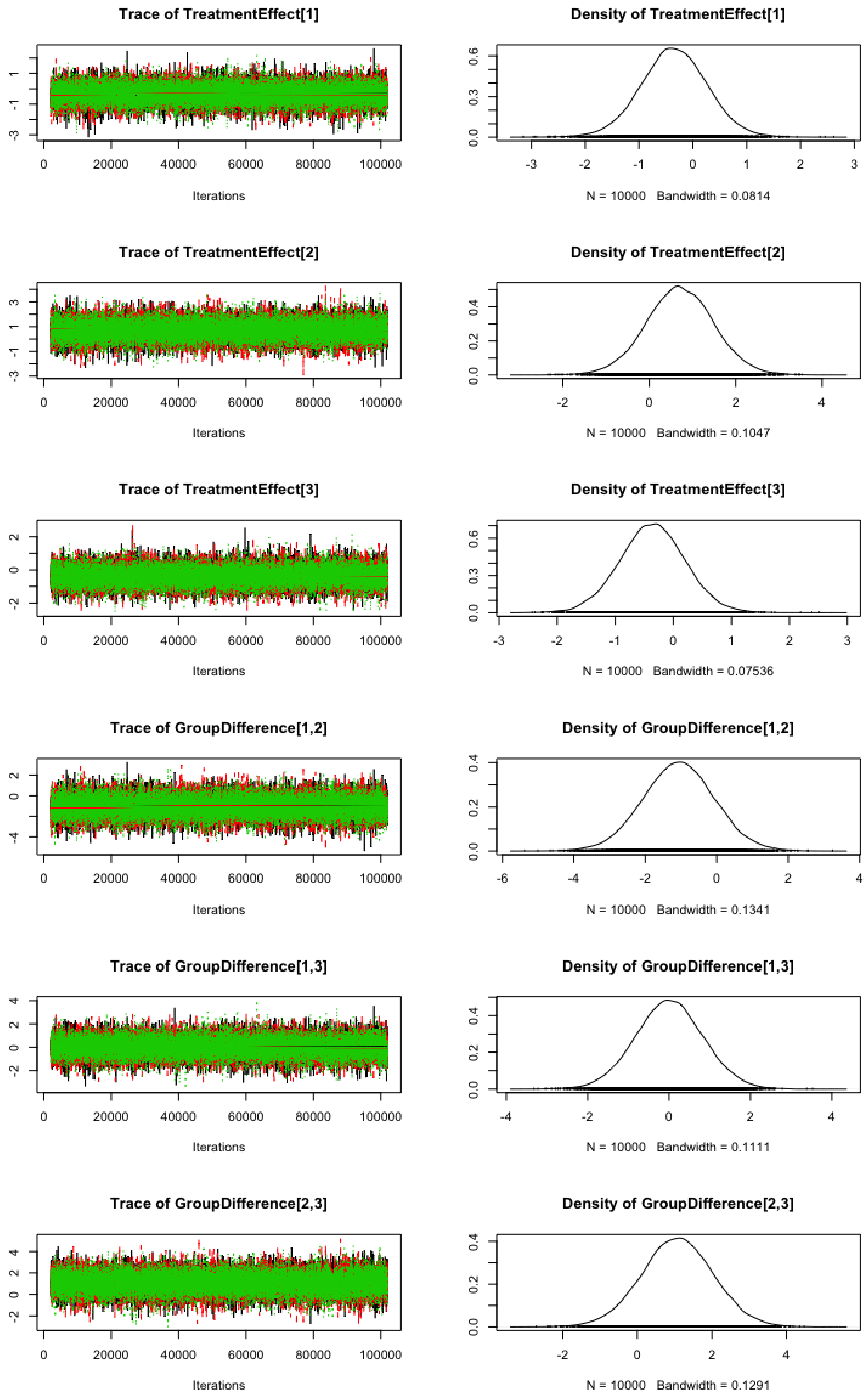

Supplementary Figure 16: Perceived Financial Self-Efficacy Sensitivity Analysis Trace and Density Plots

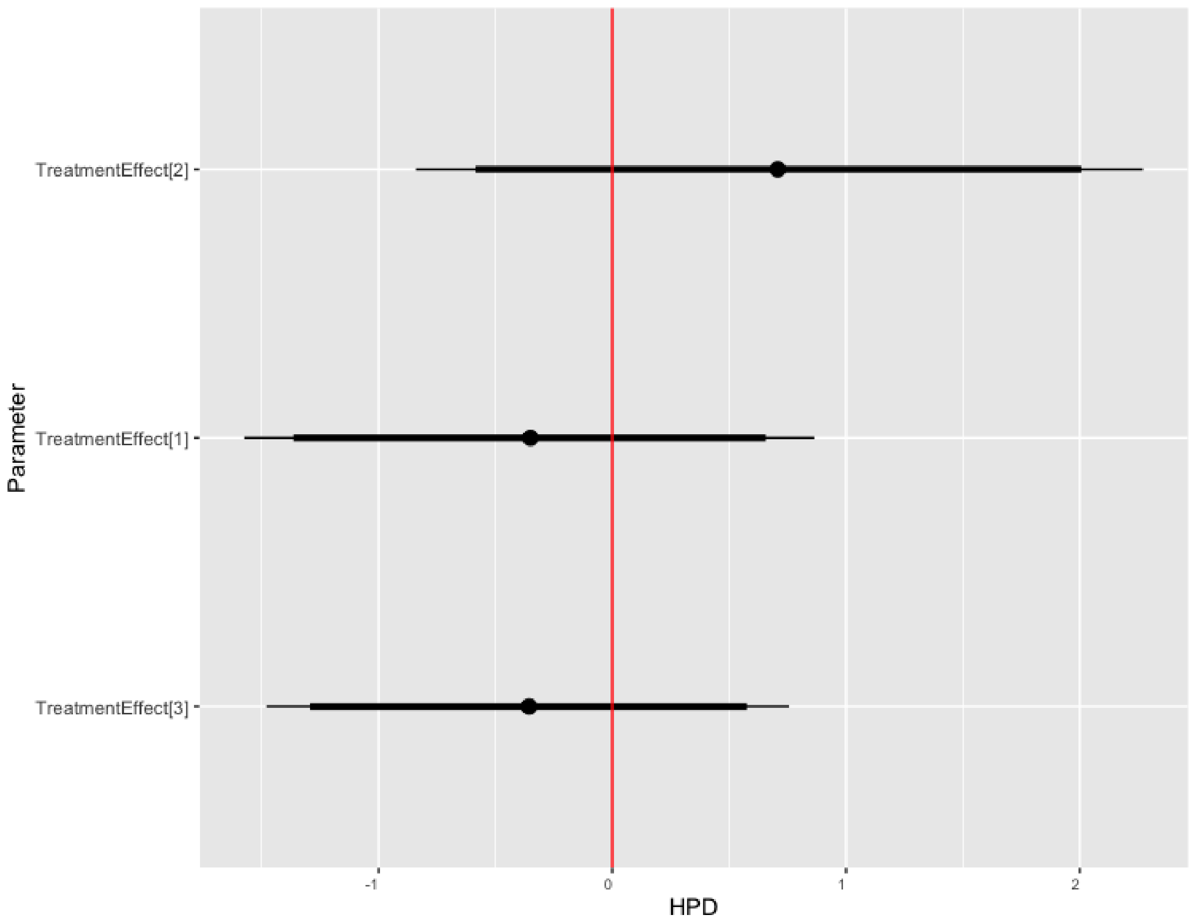

Supplementary Figure 17: Perceived Financial Self-Efficacy Sensitivity Analysis Treatment Effects by Group
Note: Group [1]: Financial Education; [2]: Design Thinking; [3]: Numeracy
HPD: Highest Posterior Density; solid black dots indicate posterior means; bold black lines represent a 90% HPD; error bars indicate a 95% HPD

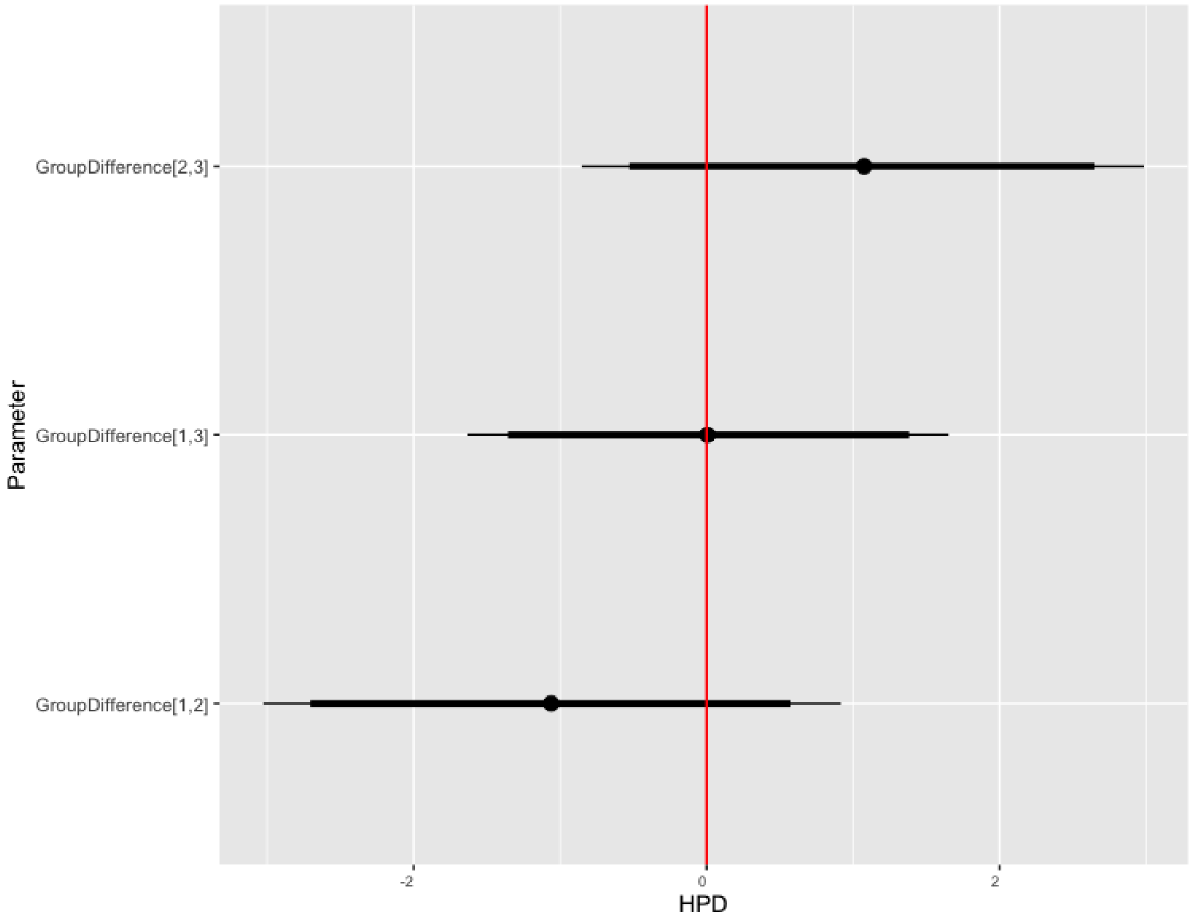

Supplementary Figure 18: Perceived Financial Self-Efficacy Sensitivity Analysis Group Differences
Note: Group [1]: Financial Education; [2]: Design Thinking; [3]: Numeracy
HPD: Highest Posterior Density; solid black dots indicate posterior means; bold black lines represent a 90% HPD; error bars indicate a 95% HPD

Bayesian Proportion Tests for Choice of Immediate vs Delayed Payment

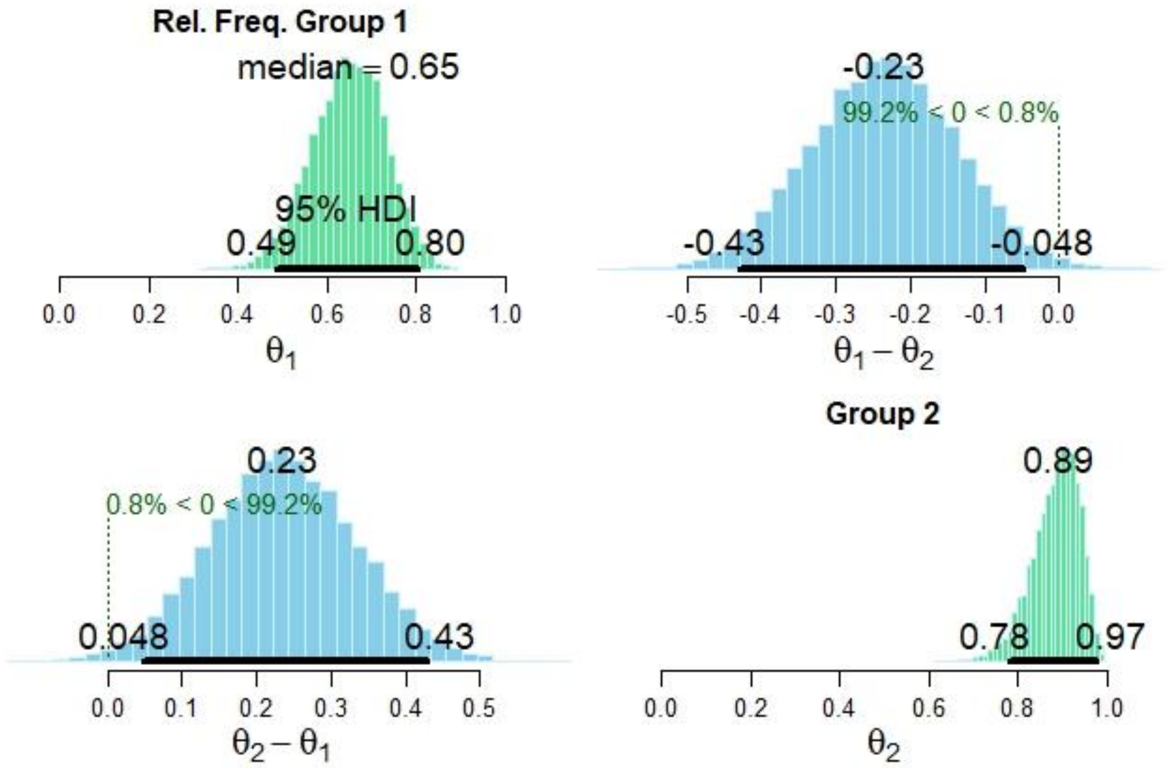

Supplementary Figure 19: Financial Education vs Design Thinking Mean Differences
Note: Group 1 Financial Education, Group 2 Design Thinking

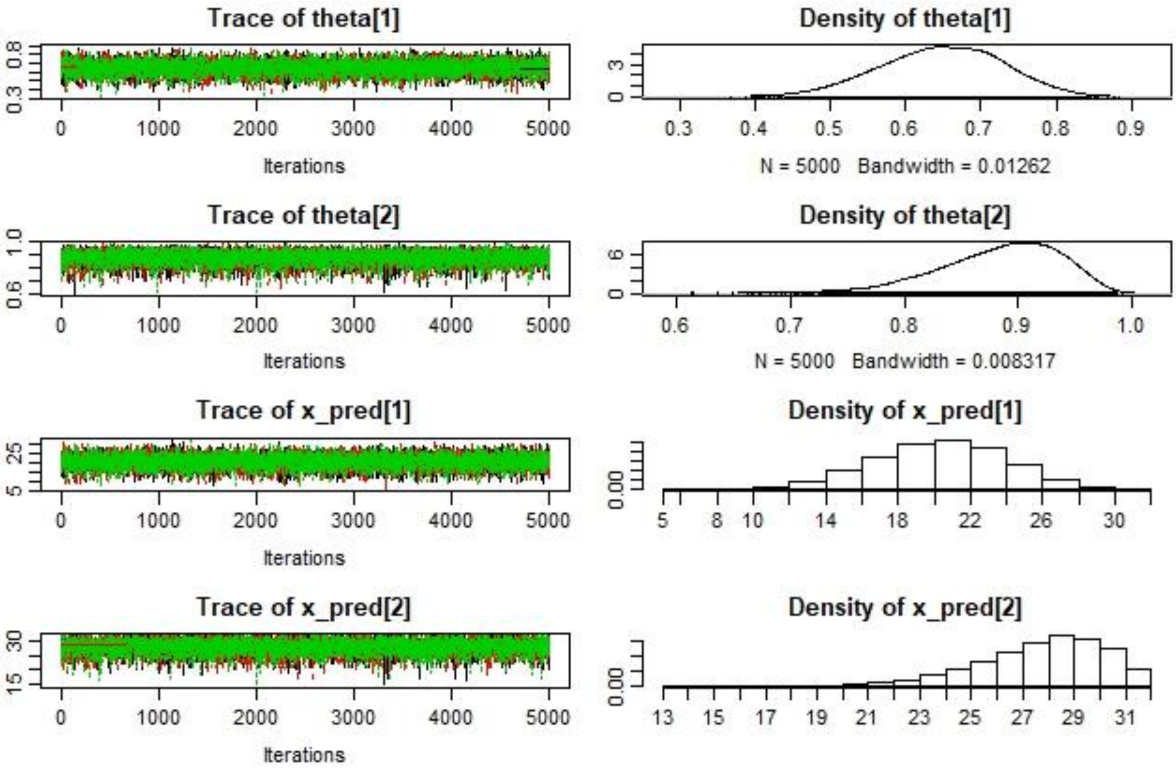

Supplementary Figure 20: Financial Education vs Design Thinking Trace and Density Plots
Note: Group 1 Financial Education, Group 2 Design Thinking

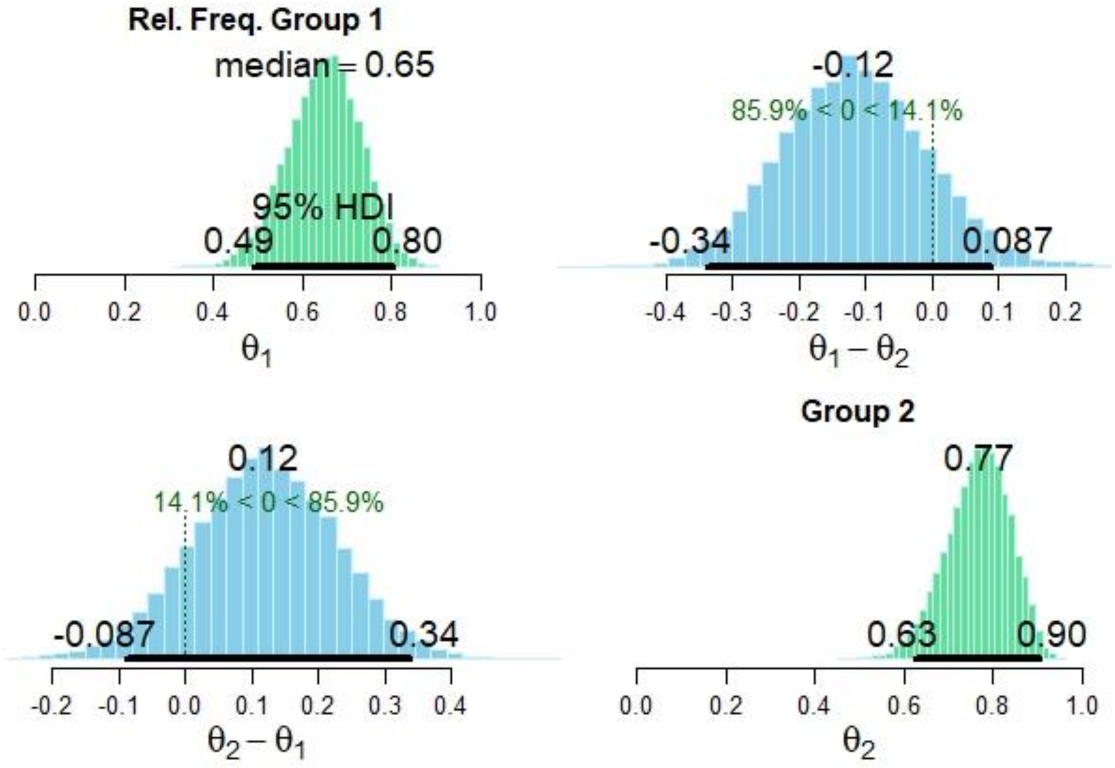

Supplementary Figure 21: Financial Education vs Numeracy Mean Differences
Note: Group 1 Financial Education, Group 2 Numeracy

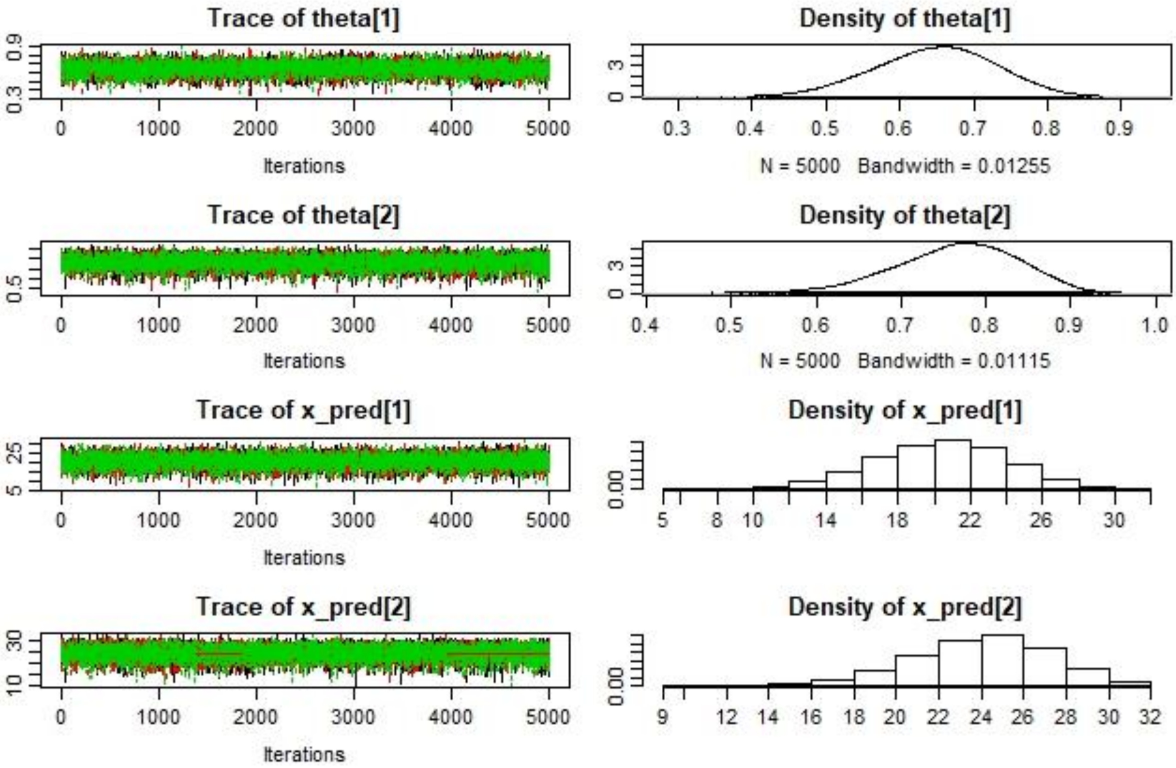

Supplementary Figure 22: Financial Education vs Numeracy Trace and Density Plots
Note: Group 1 Financial Education, Group 2 Numeracy

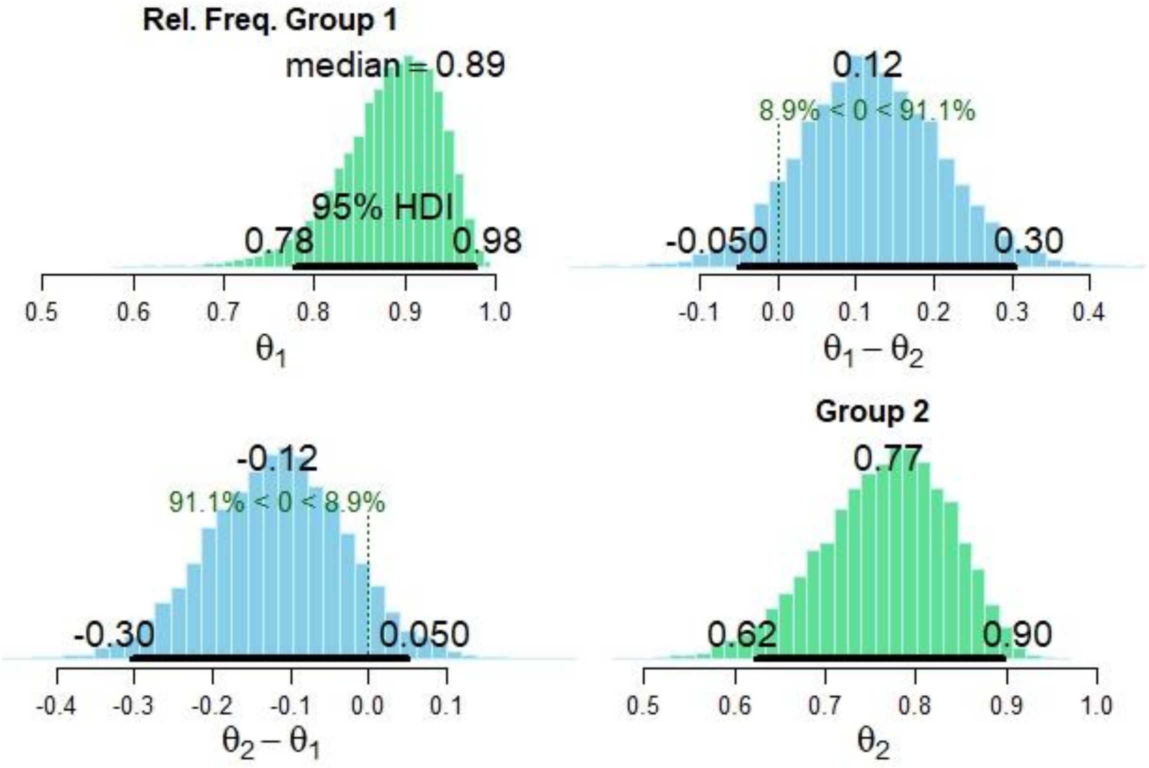

Supplementary Figure 23: Design Thinking vs Numeracy Mean Differences
Note: Group 1 Design Thinking, Group 2 Numeracy

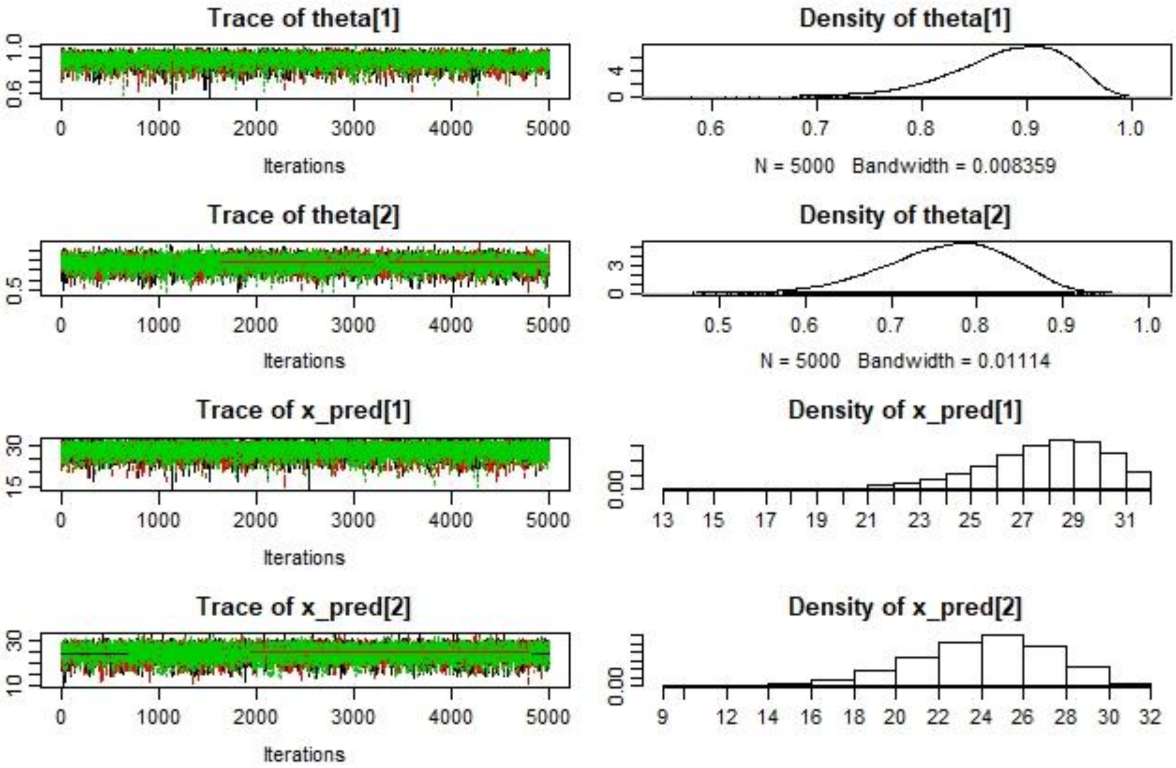

Supplementary Figure 24: Design Thinking vs Numeracy Trace and Density Plots
Note: Group 1 Design Thinking, Group 2 Numeracy
